# Nuclear factor I-C overexpression promotes monocytic development and cell survival in acute myeloid leukemia

**DOI:** 10.1038/s41375-022-01801-z

**Published:** 2022-12-26

**Authors:** Namrata Rastogi, Juan Bautista Menendez Gonzalez, Vikas Kumar Srivastava, Bader Alanazi, Rehab N. Alanazi, Owen M. Hughes, Niamh S. O’Neill, Amanda F. Gilkes, Neil Ashley, Sumukh Deshpande, Robert Andrews, Adam Mead, Neil P. Rodrigues, Steve Knapper, Richard L. Darley, Alex Tonks

**Affiliations:** 1 Department of Haematology, Division of Cancer & Genetics, School of Medicine, Cardiff University, Cardiff, CF14 4XN Wales UK; 2 European Cancer Stem Cell Research Institute, School of Biosciences, Cardiff University, Cardiff, CF24 4HQ Wales UK; 3 Department of Stem Cell and Regenerative Biology, Harvard Stem Cell Institute, Harvard University, Cambridge, MA USA; 4 Division of Infection and Immunity, School of Medicine, Cardiff University, Cardiff, CF14 4XN UK; 5 Prince Mohammed Medical City, AlJouf, Saudi Arabia; 6 Research Center, King Fahad Medical City, Riyadh, Saudi Arabia; 7 Medical Laboratory Technology Department, College of Applied Medical Sciences, Northern Border University, Arar, 91431 Saudi Arabia; 8 Cardiff Experimental and Cancer Medicine Centre (ECMC), School of Medicine, Cardiff University, Cardiff, CF14 4XN Wales UK; 9 Haematopoietic Stem Cell Biology Laboratory, Medical Research Council Weatherall Institute of Molecular Medicine, University of Oxford, Oxford, OX3 9DS UK; 10 Present Address: Department of Haematology, Division of Cancer & Genetics, School of Medicine, Cardiff University, Cardiff, CF14 4XN Wales UK

**Keywords:** Acute myeloid leukaemia, Cell signalling, Cancer

## Abstract

Nuclear factor I-C (NFIC) belongs to a family of NFI transcription factors that binds to DNA through CAATT-boxes and are involved in cellular differentiation and stem cell maintenance. Here we show NFIC protein is significantly overexpressed in 69% of acute myeloid leukemia patients. Examination of the functional consequences of NFIC overexpression in HSPCs showed that this protein promoted monocytic differentiation. Single-cell RNA sequencing analysis further demonstrated that NFIC overexpressing monocytes had increased expression of growth and survival genes. In contrast, depletion of NFIC through shRNA decreased cell growth, increased cell cycle arrest and apoptosis in AML cell lines and AML patient blasts. Further, in AML cell lines (THP-1), bulk RNA sequencing of NFIC knockdown led to downregulation of genes involved in cell survival and oncogenic signaling pathways including mixed lineage leukemia-1 (MLL-1). Lastly, we show that NFIC knockdown in an ex vivo mouse MLL::AF9 pre-leukemic stem cell model, decreased their growth and colony formation and increased expression of myeloid differentiation markers Gr1 and Mac1. Collectively, our results suggest that NFIC is an important transcription factor in myeloid differentiation as well as AML cell survival and is a potential therapeutic target in AML.

## Introduction

Acute myeloid leukemia (AML) is characterized by the rapid accumulation of a dominant clone of immature cells in the peripheral blood (PB) and bone marrow (BM) resulting in the failure of normal hematopoiesis [1]. AML is highly heterogeneous with poor clinical outcomes, averaging around 50% survival at 5 y in younger patients and 15% for those over 60 y [2, 3]. The targeted treatment of acute promyelocytic leukemic (APL) with retinoic acid-based therapy is a paradigm for successful treatment of this disease (>90% survival) [4]. Recent advancement in targeted approaches and successful approvals of potential drug candidates for AML treatment have shown promise to improve clinical outcomes of AML patients [5].

AML is characterized by a developmental arrest and is mediated through misregulation of the differentiation program. Given the prevalence of nuclear protein abnormalities in AML, we recently analyzed the nuclear proteome of AML blasts in comparison with normal human stem and progenitor cells (CD34^+^-HSPC) using 8-channel isobaric tagging (iTRAQ) coupled with tandem mass spectrometry (MS) [6]. Among already known proteins dysregulated in AML including WT1, CEBPA, we identified overexpression of several novel abnormalities including nuclear factor I-C (NFIC). NFIC belongs to the NFI family of transcription factors which includes NFIA, NFIB, and NFIX [7]. Regulation of cellular differentiation is reported to be the fundamental function of these members. For example, NFIC can induce differentiation of osteoblasts and odontoblasts [8, 9]. In solid tumors, NFIC has been shown to be upregulated in lung squamous cell carcinoma, colorectal cancer, as well as gastric cancer and is correlated with increased expression of oncogenes [10–13]. Conversely, its downregulation has been associated with increased epithelial to mesenchymal transition and metastasis in breast, bladder, and esophageal squamous cell cancer [14–16]. However, the significance of its expression in normal human hematopoietic development and the functional implication of altered NFIC expression in AML remain unidentified.

In this study, we show that NFIC overexpression promotes monocytic differentiation and increases cell survival. Conversely, loss of NFIC expression leads to cycle arrest and non-apoptotic cell death suggesting that AML cells are dependent on the expression of this protein for their survival.

## Materials and methods

### Primary samples and cell culture

Human neonatal cord blood was obtained with approval from the South-East Wales Research Ethics Committee from healthy full-term pregnancies at the University Hospital of Wales, Cardiff, UK. Normal human cord blood-derived CD34^+^ (>90% purity) HSPCs were isolated and cultured as previously described [6, 17]. Diagnostic AML BM or PB samples were obtained from patients enrolled in the NCRI AML trials (Supplementary Table S1). All primary material was collected following informed consent and processed in accordance with the 1964 Declaration of Helsinki. AML cell lines were obtained from ATCC (Middlesex, UK) or ECACC^TM^ (Salisbury, UK) and cultured under recommended culture conditions (Supplementary Materials and methods).

### Generation of control and NFIC expressing/knockdown human myeloid progenitor cells

Short hairpin RNA (shRNA) vectors with GFP or-m-Cherry selectable markers were designed and purchased from VectorBuilder (Guangzhou, China) (Supplementary Materials and methods). Lipofectamine 3000 was used to generate lentivirus through transient transfection of HEK293 packaging cells according to the manufacturer’s instructions (Fisher Scientific, Loughborough, UK). Normal human CD34^+^ HSPC and cell lines were transduced with lentivirus as previously described [6, 18]. Following infection (day 3 of culture), cells were maintained in bulk-liquid culture for growth and differentiation assessment by flow cytometry (see “Hematopoeitic differentiation”).

### Hematopoietic differentiation and colony-forming assay

Following transduction human CD34^+^ HSPCs were cultured in Iscove’s Modified Dulbecco’s Medium (IMDM; Fisher Scientific, Loughborough, UK) supplemented with the following cytokines at 5 ng/mL and incubated at 37 °C with 5% CO_2_ in air for 9 days: IL-3, SCF, G-CSF and GM-CSF (BioLegend, London, UK). Hematopoietic differentiation was assayed by flow cytometry at days 3, 6, and 9 of culture for monocytic, granulocytic and erythrocytic lineages using cell surface markers as previously described [18].

For myeloid colony formation assay, transduced normal human HSPCs cells were FACSorted for GFP^+^. Subsequently cells were seeded by limiting dilution (0.3 cells/ well) in 96-U well plates in IMDM medium described above. Myeloid colonies (and clusters) were counted at day 14 of culture. Colony replating was performed by pooling colonies from 14-day cultures and re-seeded at limiting dilution (1 cell/well) for a further 14 days. Replated colonies were again counted and colony-forming efficiency was calculated. For colony formation in semi-solid medium MLL::AF9 transformed pre-LSCs were infected with shRNA or scrambled control, sorted for GFP^+^/m-Cherry^+^ cells as previously described [19]. 2000 sorted cells were seeded in methylcellulose-containing media MethoCult M3231 supplemented with SCF (20 ng/mL), IL-3 and IL-6, and GM-CSF (10 ng/mL) (STEMCELL Technologies, Cambridge, UK) following the manufacturer’s instructions.

### Migration assay

Transduced CD34^+^ HSPCs cells were seeded on top of trans-well inserts (Corning, NY, USA) placed within 24-well culture plates containing the following chemotactic growth medium; IMDM + 10% *v/v* FBS + SDF-1 (Peprotech, London, U.K.) and incubated at 37 °C for 6 h. Following incubation cells were harvested and GFP^+/−^ cells counted using flow cytometry to calculate percent GFP^+^ migration.

### Cell proliferation, cell cycle, and apoptosis assays

Transduced cells were seeded at a density of 2 × 10^5^ cells per well and viable cell counts were determined daily using 7-Actinomycin D (7-AAD, 10 μg/mL). Following five days of culture, cells were harvested, washed with PBS, and fixed with 70% *v/v* ethanol on ice for 30 min. Fixed cells were washed and stained with propidium iodide (PI) 50 μg/mL and RNAseA (100 μg/mL) and analyzed by flow cytometry. Cells were also stained with Annexin-V-PE and 7-AAD using apoptosis detection kit (Cambridge Biosciences, UK).

### Western blotting

Cytosolic and nuclear protein lysates were extracted and immunoblotted as previously described (Supplementary Materials and methods) [6]. Primary antibodies used included NFIC anti-human (1C12-2A2; Bio-Techne Ltd, Abingdon, UK), Nfic anti-mouse (ABE1387; Sigma), Histone H1 (clone AE-4; AbD Serotec, UK), glyceraldehyde-3-phosphate dehydrogenase (GAPDH, Santa Cruz Biotechnology, Heidelberg, Germany), β-actin (AC-15, Sigma) and apoptotic inducing factor (AIF, B-9, SC). HRP-conjugated secondary antibodies were purchased from GE Healthcare, UK.

### RNA sequencing

For single-cell RNA sequencing viable GFP^+^7-AAD^−^ human CD34^+^ HSPCs were FACsorted following four days of transduction. 10,000 sorted cells per sample were processed for single-cell nuclei for RNA sequencing according to manufacturer’s instruction (10× Genomics, Pleasanton, CA). Briefly Gel-Beads in emulsion (GEM) were generated using Chromium Next GEM Single Cell 3′GEM Kit v3.1 (10x Genomics). cDNA libraries were constructed using Chromium Next GEM Single Cell 3′ Library Kit v3.1 (10x Genomics) and sequenced using Illumina NextSeq 500 system using the NextSeq 500/550 High Output v2 Kit (Illumina, San Diego, CA). Data from 10X Genomics was analyzed using CellRanger software v.3.1.0. as well as Partek Flow single cell analysis module (Version: 6.0.17.1206) for cellular heterogeneity and differential gene expression. For bulk mRNA sequencing THP-1 cells transduced with NFIC shRNA or control (GFP only) vector were sorted for GFP, four days after transduction. Total RNA was isolated using All Prep DNA/RNA kit (Qiagen GmBH, Hilden, Germany) according to the manufacturer’s protocol. Samples with RNA integration number (RIN) of >9 was used for sequencing analysis using Novaseq 6000 S4. Data analysis was performed through R and R studio and pathway analysis was performed using Gene Set enrichment Analysis (GSEA) and Ingenuity Pathway Analysis (IPA) software. For detailed protocol and data analysis see Supplementary Material and methods. Raw and processed data from scRNA seq and RNA seq is available at Gene Expression Omnibus with accession numbers GSE200456 and GSE196045 respectively

### Flow cytometry

To study human HSPCs differentiation, cells post-infections were analyzed with the differentiation cocktail for 30 min at 4 °C, washed and analyzed using BD FACSCanto II and LSR Fortessa. For single-cell sequencing 7-AAD^−^ and GFP^+^ cells were sorted using FACS Aria III. Data were analyzed using FCS express software V7 (Pasadena, CA, US). Details for gating strategy and analysis are in Supplementary Material and methods.

### Statistical analysis

All data were analyzed using GraphPad Prism software (v9.0). Assuming equal variances, one-way and two-way Analysis of Variance (ANOVA), or student ‘*t*’ test were used to determined statistical significance as indicated in figure legends. Dunnett’s test was used for multiple comparisons. Mann-Whitney *U* test was used for comparing mRNA expression in patient samples. Gene expression data from the online data sets were analyzed using BloodSpot. Batch correction between different data sets was performed by RMA normalization, with AML samples of each individual group was compared to HSC or with their closest counter otherwise stated [20]. For RNA sequencing data sets, False Discovery Rate (FDR; adjusted *p* value) was calculated using Benjamin-Hochberg test with FDR < 0.05 was considered as a significant. For GSEA analysis gene sets with a cut off nominal *p* value <0.05 and FDR of <0.25 was considered significant.

## Results

### NFIC is overexpressed in AML blasts

**Fig. 1:**
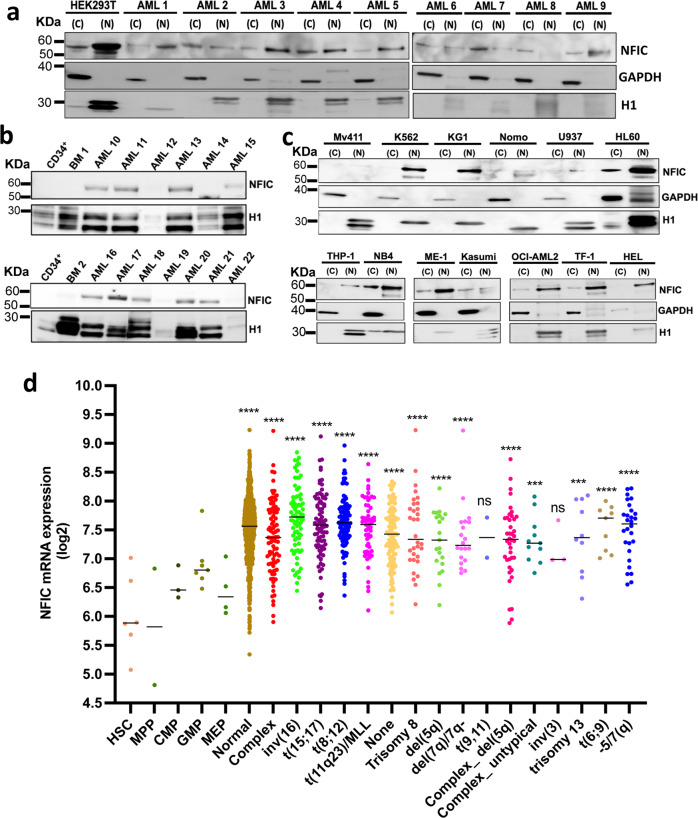
NFIC is overexpressed in AML. **a** Immunoblot showing NFIC (~56KDa) protein expression in subcellular fractions of FAB-M1 AML patient blasts. NFIC overexpressing HEK-293T cells (Supplementary Fig S1b) were used as a positive control to determine NFIC expression. GAPDH and Histone 1 (H1) were used as loading controls for cytosolic (C) and nuclear (N) protein respectively. **b** Immunoblot of NFIC protein expression in AML patient blasts compared to CD34^+^ HSPCs and normal human bone marrow (BM) in nuclear lysates in a second independent patient cohort to that of samples previously analyzed by MS. **c** NFIC protein expression in AML cell lines. **d** *NFIC* mRNA expression (Log_2_) in Normal hematopoietic subsets and in different AML subtypes (For complete description of AML subtypes and normal blood cells refer to Bloodspot data set for BloodPool: AML samples with normal cells) and normal hematopoietic cells analyzed using BloodSpot and the following data sets: GSE13159, GSE15434, GSE61804, GSE14468, The Cancer Genome Atlas (TCGA) and GSE42519 (for normal blood cells) [20–28] Horizontal markers indicate median expression intensities. Tukey’s test was used to analyze the level of statistical significance of individual AML subtype compared to normal HSC where ***p < 0.001, ****p < 0.001 and ‘ns’ is non-significant.

Initially, we validated NFIC protein expression by western blot in HEK cells transfected for NFIC (Supplementary Fig. S1). Further, we were unable to detect significant NFIC protein expression in normal human HSPCs or normal human BM MNCs (Supplementary Fig. S1). We previously identified overexpression of NFIC in the nucleus of 40% (6/15) of FAB-M1 AML patients (>5-fold compared to normal HSPC based on relative peptide frequencies) [6]. To support the upregulation of NFIC that we identified by iTRAQ MS, we examined nuclear NFIC protein expression by western blotting and confirmed overexpression in 8/9 AML samples analyzed by MS. Further, all these samples also showed aberrant cytosolic expression of NFIC (Fig. 1a). In contrast, we next extended this analysis using an independent cohort of 13 random samples from AML patients and found nuclear expression of NFIC in 69% (9/13) of patient samples (Fig. 1b). Further, nuclear NFIC protein expression was observed in 9/13 (69%) AML cell lines (Fig. 1c).

To determine whether the overexpression was transcriptionally driven we used the corresponding transcriptomic from patients used in our MS studies [6]. We found *NFIC* mRNA expression (log_2_) to be significantly upregulated by 1.5-fold in AML compared to normal HSPC (Supplementary Fig. S2a). Analysis of publicly available transcriptomic data sets [21–29] also showed *NFIC* mRNA was significantly upregulated in AML vs HSC (Fig. 1d and Supplementary Fig. S2b). Taken together, these data suggest that AML cells have high expression of NFIC compared to normal HSPCs.

### NFIC overexpression promotes growth and monocytic development

**Fig. 2:**
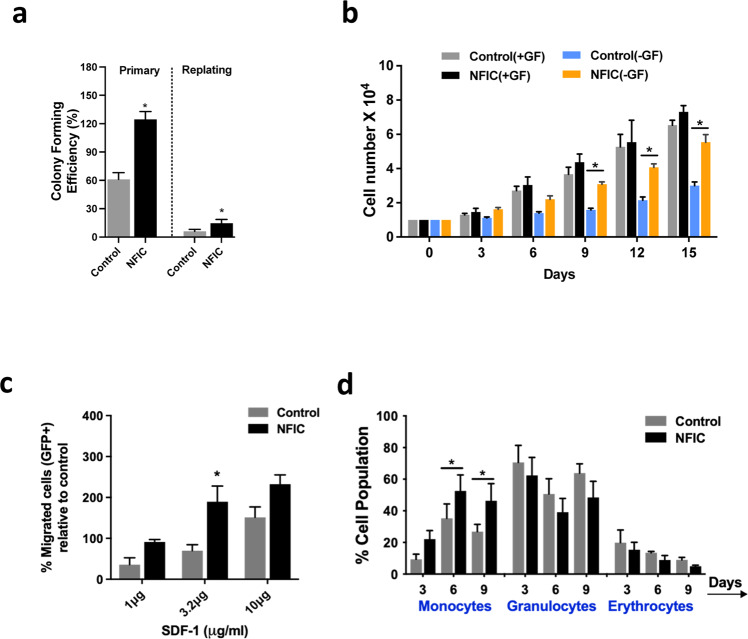
NFIC overexpression disrupts normal hematopoiesis. **a** Bar graph represents average number of primary and secondary granulo-myelocytic (GM) colonies in NFIC overexpressed (NFIC) vs GFP only control. Infected CD34^+^ HSPCs were sorted for GFP three days post infection and seeded at a density of single cell per 3 wells in 96 well plates for colony formation assay. Myeloid colonies were counted after 14 days of culture. For replating assay, primary colonies were pooled, counted, and re-seeded by limiting dilution (1 cell/well) in 96 well plates and counted after 14 days. Data represents mean ± 1 SD (*n* = 3). Statistical significance was analyzed using Dunnett’s multiple comparison test with *p < 0.05 **b** Total cell counts represented as bar graphs. CD34^+^ HSPCs expressing NFIC or Control were sorted on day 3 for GFP^+^ and seeded in 24-well plate in IMDM with growth factor (+GF) or without growth factors (−GF) for cell growth and survival assay. Live cells were counted using trypan blue stain at indicated days. Data represents mean ± 1 SD (*n* = 3). Statistical significance was analyzed using Dunnett’s multiple comparison test with *p < 0.05. **c** GFP^+^ CD34^+^ HSPCs at 3 days post infection were seeded on top of transwell inserts in 24-well plates containing chemotactic media with SDF-1 at indicated concentrations. Migration response was calculated as the percentage of GFP-positive cells that migrated across the transwell membrane. Data represents mean ± 1 SD (*n* = 3). **d** Bar graphs represents percentage cell populations of monocytes, granulocytes, and erythrocytes at 3, 6, and 9 day of culture. CD34^+^ HSPCs, expressing NFIC or GFP only (Control) were seeded in cytokine-rich media to study differentiation at indicated days. Cell surface differentiation markers on GFP-gated cells were used to identify different cellular types (details in the Supplementary methods). Data represent mean ± 1 SD (*n* = 3). Statistical significance was analyzed using Dunnett’s multiple comparison test with test with *p < 0.05.

Higher expression of NFIC in AML prompted us to determine the effects of NFIC overexpression on colony formation and growth of normal HSPCs. We overexpressed NFIC in CD34^+^ HSPCs using a vector co-expressing NFIC and GFP (Supplementary Fig. S3a) and determined the effects of NFIC overexpression on myeloid colony forming capacity under clonal conditions. Overexpression of NFIC significantly increased the number of colonies by 2-fold compared to control (Fig. 2a). To gauge the impact of NFIC overexpression on replating potential we carried out a secondary replating of colony-forming cells. NFIC overexpressing cells were able to form 2.4-fold more myeloid colonies than control. Given that NFIC promoted myeloid colony formation, we next determined whether NFIC overexpression affected growth of normal HSPCs under normal and cytokine-deprived culture conditions. NFIC overexpression did not significantly impact the growth of HSPCs in cytokine-supplemented growth (Fig. 2b); however, we observed enhanced growth of NFIC-expressing cells under cytokine-deprived conditions where results were significant for day 9 (1.9-fold), day 12 (1.8-fold) and day 15 (1.8-fold) (Fig. 2b). Next, given that changes to cellular migration is a characteristic feature of pre-leukemic and leukemic cells [30, 31] we determined whether NFIC overexpression could affect HSPC migration (Fig. 2c). NFIC overexpression significantly promoted migration to SDF-1 by up to 3.5-fold compared to controls. Lastly, to determine whether NFIC overexpression can perturb normal human hematopoietic development, we analyzed the proportion of monocytes, granulocytes and erythrocytes that were derived from CD34^+^ HSPC over time. Overexpression of NFIC significantly increased the proportion of monocytes by 1.7-fold on day 9 of culture compared to control, possibly at the expense of granulocyte development. These data suggest that NFIC overexpression may promote monocytic differentiation (Fig. 2d).

Taken together, these data suggest that NFIC overexpression promotes monocytic development and myeloid colony formation coupled with enhanced cytokine-independent growth and migration.

### scRNA seq reveals NFIC-mediated skewing of hematopoiesis through increased number of monocytes with dysregulated gene expression profiles

**Fig. 3:**
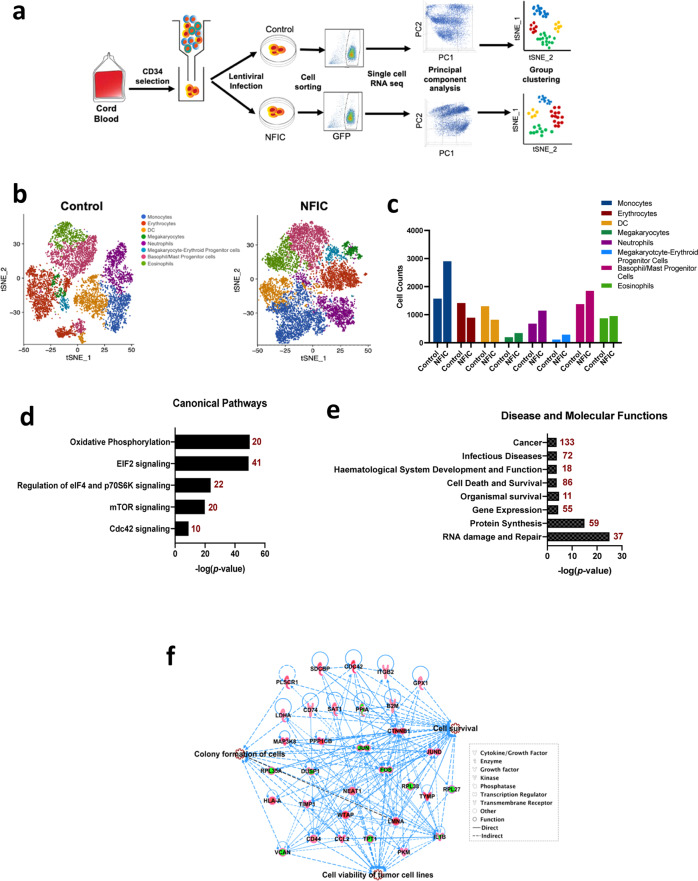
Single-cell RNA sequencing reveals increases in numbers and changes in cellular gene expression of NFIC overexpressing monocytes. **a** Graphical representation of single-cell mRNA sequencing of NFIC overexpressing normal blood cells. 8000 GFP^+^ sorted cells were processed for nuclei isolation, library preparation, and single-cell RNA sequencing using Chromium 10x genomics. **b** t-distributed stochastic neighbor (t-SNE) embedding analysis for representing different cell clusters identified based on principal component analysis (PCA). Cells are colored on the basis of different cell type clusters formed. **c** Bar graph represents total cell numbers within each indicated cell type clusters in GFP only (Control) vs NFIC overexpressing (NFIC) group. **d** Differential gene expression analysis between NFIC overexpressing monocytes *vs* control identified top canonical pathways and **e** disease and molecular function terms enriched after differential gene expression within the monocytes cell cluster using IPA. Each bar graph represents the cellular pathway and x-axis represents the −log_10_(*P*) values which determine the level of significance and numbers besides each bar shows number of genes altered in within that pathway. **f** Network map for all significant genes associated within cell survival pathway with three most enriched functional nodes and their interconnections. Red shades indicate upregulation of genes whereas green shades indicate downregulation. A complete list of these genes is given in Supplementary Table 3. The network map was generated using IPA Path Designer tool. Legends show in the text box at the right of the map.

Since NFIC is a transcriptional regulator, we next determined the impact of NFIC on developmental hematopoietic subsets at a single-cell level (Fig. 3a). t-SNE plot was used to visualize eight cell clusters which were identified based on the top cellular markers expressed within each cluster (Fig. 3b and Supplementary Fig. S3b, c). We found increase in numbers of cells of monocytic (1.8-fold), neutrophils (1.7-fold) and basophil/mast progenitor (1.3-fold) lineage cells after NFIC overexpression compared to control, however, increase in numbers of monocytic lineage cells was more pronounced as compared to other lineages (Fig. 3c). Therefore, to understand the molecular basis of this, we focussed our analysis on monocytes to determine changes in mRNA expression following NFIC overexpression. We identified 164 and 88 significantly upregulated and downregulated genes, respectively (*p* < 0.05, FDR < 0.05) in NFIC overexpressing monocytes as compared to control (Supplementary Table S2). Pathway analysis identified enrichment in genes involved in mTOR signaling, cancer cell death and survival, energy metabolism pathways and oxidative phosphorylation (see “Discussion”) (Fig. 3d). This analysis was supported by an enrichment analysis for disease and molecular functions, further demonstrating a high number of genes enriched for cancer and cell death and cell survival pathways (Fig. 3e). To further explore the mechanistic pathway of cell death and cell survival we created a network analysis map using all the genes enriched within this mechanistic network using IPA Build tool. We found that most of the genes associated with cell survival were found to be upregulated in NFIC overexpressing as compared to control monocytes (Supplementary Table S3) and were sub-divided into three major functional nodes of cell survival, colony formation and cell viability of tumor cells (Fig. 3f). These results support the above data in that NFIC overexpression in HSPCs skews normal hematopoiesis towards increased monocytic development.

### AML cell survival is dependent on NFIC expression

**Fig. 4:**
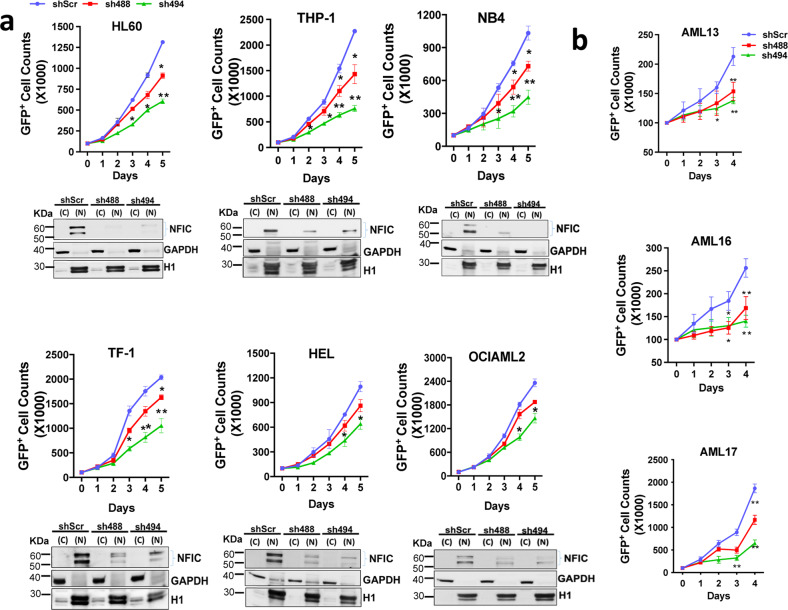
NFIC knockdown reduces growth of AML cells. **a** Line graphs represent total cell counts at different days post NFIC knockdown (KD). AML cell lines HL60, THP-1, NB4, TF-1, HEL and OCIAML2 were infected with either of the two NFIC shRNAs (sh488 and sh494) or scrambled shRNA (shScr). Forty-eight hours post-infection cells were seeded in 24-well plates designated as Day 0. GFP^+^ viable (7-AAD) cells were counted every 24 h for up to 5 days of culture. Immunoblots below each graph show KD of NFIC protein compared to control. GAPDH and Histone H1 were used as loading controls for cytosolic (C) and nuclear (N) extracts respectively. Data is represented as mean ± 1 SD (*n* = 3). **b** Line graphs showing total GFP^+^ cell counts of primary AML patient-derived blasts infected with shRNA or control. Forty-eight hours post-infection cell were seeded and GFP^+^ cell counts were performed every 24 h for four days. KD efficiency of shRNAs was also evaluated through intracellular staining and flow cytometric analysis (Supplementary Fig S4b). Data represented are mean ± 1 SD (*n* = 3). Statistical significance denoted with *p < 0.05 and **p < 0.01 when analyzed by Dunnett’s multiple comparison test.

Data above show that ectopic expression of NFIC perturbs normal hematopoiesis and promoted the growth of normal human HSPCs under cytokine-deprived conditions. Therefore, we next determined if AML cells were dependent on NFIC expression for growth and survival. We selected cell lines with high expression of NFIC (Fig. 1) and transduced them with different shRNA targeting this gene. Five different shRNAs were screened for NFIC knockdown (KD) of which two with the highest KD efficiency were used in subsequent studies (Supplementary Fig S4a). NFIC KD with sh494 reduced the growth of HL60 (0.5-fold), THP-1 (0.4-fold), NB4 (0.4-fold), TF1 (0.5-fold), HEL (0.6-fold) and OCIAML2 (0.6-fold) cells at day 5 as compared to scrambled control (Fig. 4a). Further, KD of NFIC in primary AML blasts (Supplementary Fig S4b) also significantly reduced growth ranging from 0.6-fold (sh488 vs Scr) and 0.3-fold at day 4 (Fig. 4b).

**Fig. 5:**
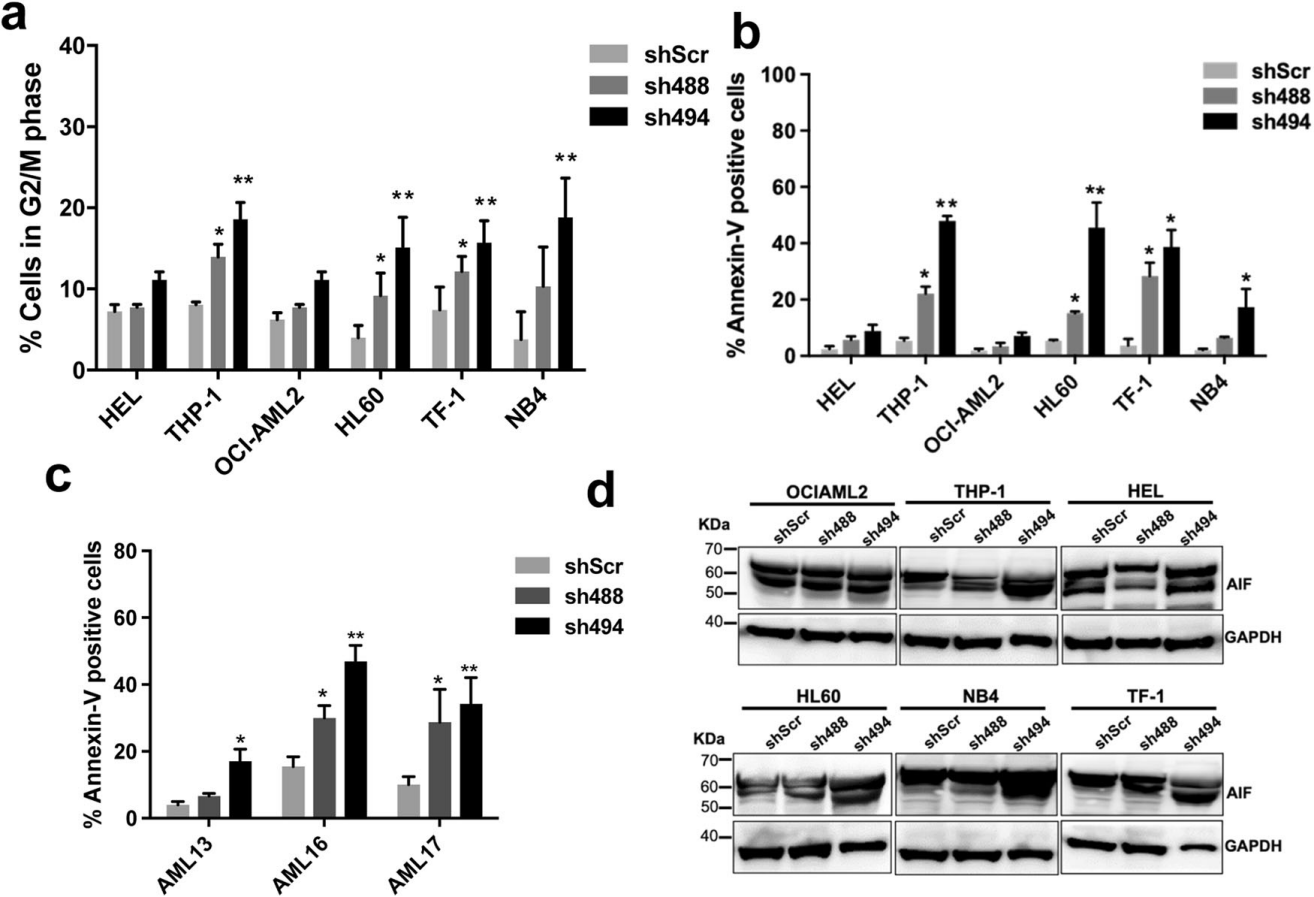
NFIC knockdown reduces survival of AML cells by inducing apoptosis and cell cycle arrest. **a** Cell cycle analysis of NFIC KD AML cell lines. Bar graphs represent percentage of cells in G2/M cell cycle phase. Cell cycle analysis was performed after four days of infection with NFIC shRNAs as compared to scrambled RNA. Data represents mean±1 SD with *p < 0.05 and **p < 0.01 as analyzed by Dunnett’s multiple comparison test. **b** Bar graph represents percentage of Annexin-V^+^ cells. AML cell lines were infected with NFIC shRNA (sh488 and sh494) or scrambled shRNA (shScr) and after four days, stained with Annexin-V and 7-AAD and analyzed by flow cytometry. The percentage of Annexin-V^+^ (early apoptotic) and Annexin-V^+^/7-AAD^+^ (late apoptotic) were pooled together to represent the extent of apoptosis in these cells. Data is represented as mean±1 SD with *p < 0.05 and **p < 0.01 as analyzed by Dunnett’s multiple comparisons test. **c** Bar graph represents percentage of Annexin-V^+^ cells. Primary AML patient PBMCs were infected with NFIC shRNAs or scrambled control. After four days cells were analyzed by flow cytometry. Bar graphs represent mean±1SD with *p < 0.05 as analyzed by Dunnett’s multiple comparison test, **d** Western blots representing expression of Apoptosis-inducing factor (AIF) Mol. Wt. 66 KDa. Infection efficiency was >95% in all cases. GAPDH was used for endogenous loading control.

We next analyzed the effect of NFIC KD on cell cycle in AML cell lines. We found that NFIC KD triggered an accumulation of AML cells in the G2/M phase of cell cycle (Fig. 5a and Supplementary Fig S4c). Cell cycle arrest was further confirmed by cyclin B1 expression, a key mediator of G2/M cell cycle phase, which was found to be increased upon NFIC KD (Supplementary Fig S4d). To determine whether reduced survival could also contribute to decreased cell growth, we analyzed annexin-V/PI staining in NFIC KD AML cell lines and primary patient samples. NFIC KD with sh494 led to significant increases in apoptosis in cell lines THP-1 (ninefold), HL60 (eightfold), NB4 (eightfold) and TF-1(tenfold) (Fig. 5b) and a similar effect was observed in AML patient samples ranging from 1.6 to 4-fold (Fig. 5c). We next determined the mechanism of apoptosis in NFIC KD cells. Western blot demonstrated that apoptosis induction was not associated with PARP activation (Supplementary Fig 4e) but elevated apoptosis did correlate with the induction of a caspase-independent mechanism through induction of apoptosis-inducing factor (AIF) (Fig. 5d). To see if we could rescue NFIC KD cells from AIF induced cell death we co-knocked down NFIC and AIF in HL60 and THP-1 cells and assayed apoptosis (Supplementary Fig S5). Whilst a reduction in NFIC-induced apoptosis was observed, the effect was not significant (Supplementary Fig S5b). Taken together these results suggest that NFIC is an essential factor for growth and survival of AML cells and its inhibition can lead to caspase-independent apoptosis in these cells.

### NFIC knockdown alters gene expression of cell survival and oncogenic pathways in THP-1 cells

**Fig. 6:**
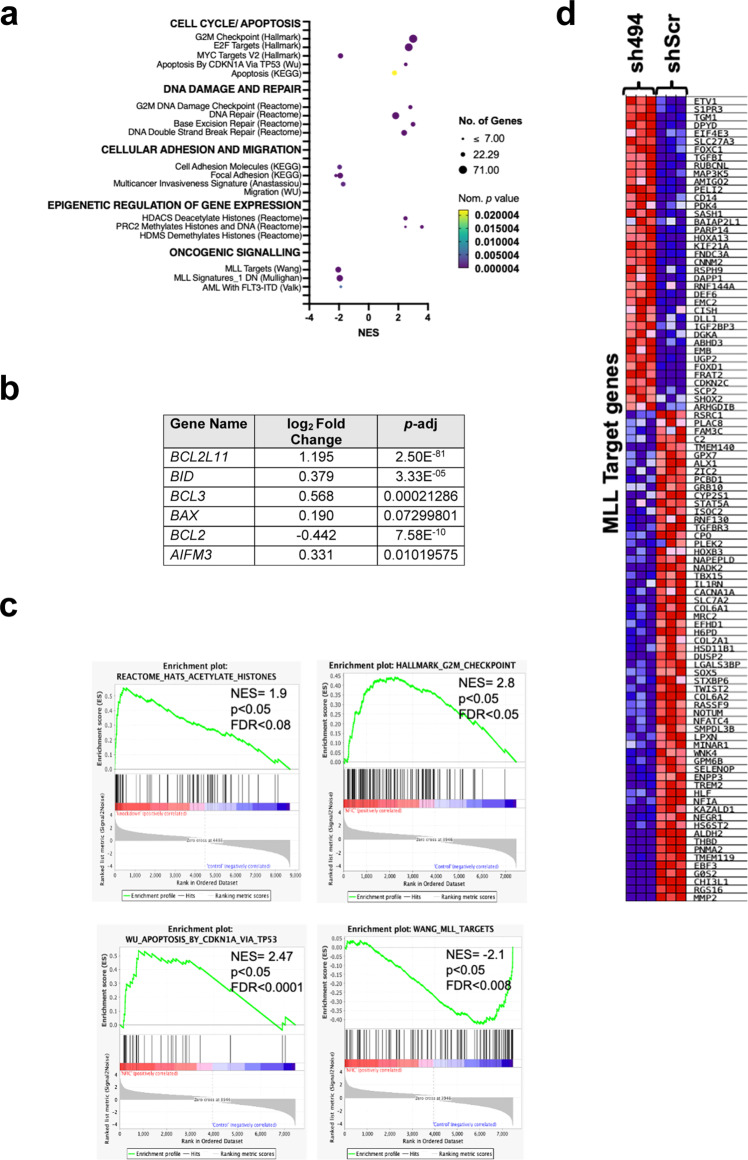
NFIC KD induces differential expression (DE) of genes in THP-1 cells. **a** Multivariable graph represents a list of pathways enriched after Gene Set Enrichment Analysis (GSEA) of all differentially expressed genes with significance (FDR < 0.05) and log_2_ fold change >1.5 or <−1.5 in THP-1 cells following NFIC KD (sh494) vs scrambled (shScr). **b** Table showing DE apoptotic-related genes in THP-1 cells following NFIC knockdown (sh494) vs scrambled (shScr). **c** GSEA shows that NFIC KD increases expression of genes involved in G2M checkpoints histone acetylation and epigenetic regulation of genes expression with a positive Normalized Enrichment Score (NES). Pathway enrichment analysis showed upregulation of genes downregulated by fusion oncogene NUP98-HOXA9 with a positive NES and downregulation of genes that are targets of MLL with a negative NES. Enriched pathway with nominal *p* < 0.05 and False Discovery Rate (FDR) < 0.05 was selected for investigation. **d** Heat map showing DEG in THP-1 cells following NFIC knockdown (sh494) vs scrambled (shScr) enriched for MLL target genes. Data represents *n* = 3 for each group.

To further understand the molecular mechanisms involved in NFIC-mediated pro-survival signaling in AML cells, we performed bulk mRNA sequencing of THP-1 cells in which NFIC was KD. PCA analysis showed distinct separation between scrambled control (C1, C2, and C4) and NFIC knockdown samples (N1, N2 and N3) indicating differential gene expression profiles between the groups (Supplementary Fig S6a). NFIC KD resulted in 10,083 significantly differentially expressed genes (Supplementary Fig S6b). GSEA analysis identified upregulation of genes involved in G2M cell cycle regulation, apoptosis, DNA repair and epigenetic regulation of gene expression as well as downregulation of genes involved in cell survival such as Myc targets, MLL targets, cell adhesion, migration, and invasion (Fig. 6a). We observed significant changes in mRNA expression of AIF (*AIFM3*) (Fig. 6b). Given that Bcl2 family proteins have shown to regulate AIF mediated caspase-independent apoptosis we looked for changes in these genes and found significant changes in expression of *BID, BAX* and *BCL2L11* (Fig. 6b). These findings suggest that AIF is not solely responsible for NFIC inhibition induced cell death in AML cells. Interestingly, we also found downregulation of MLL target genes which were upregulated after MLL-1 overexpression in mouse embryonic cells [32] (Fig. 6c, d). These results suggest a corelation of NFIC with MLL signaling in THP-1 cells. Similarly, pathway analysis using IPA also showed enrichment of genes involved in growth, survival, and migration of tumor cells (Supplementary Fig S6c). GO analysis of the genes which were significantly upregulated in NFIC knockdown THP-1 cells using Metascape showed an enrichment of biological pathways of epigenetic regulation of gene expression, differentiation, metabolism as well as cell killing (Supplementary Fig S6d). Taken together, these data suggest that NFIC may have a prominent role in cell cycle, growth and apoptosis, epigenetic regulation of gene expression, and oncogenic transformation of AML cells.

### NFIC inhibition decreased growth, survival and colony formation of MLL::AF9 pre-leukemic cell models

**Fig. 7:**
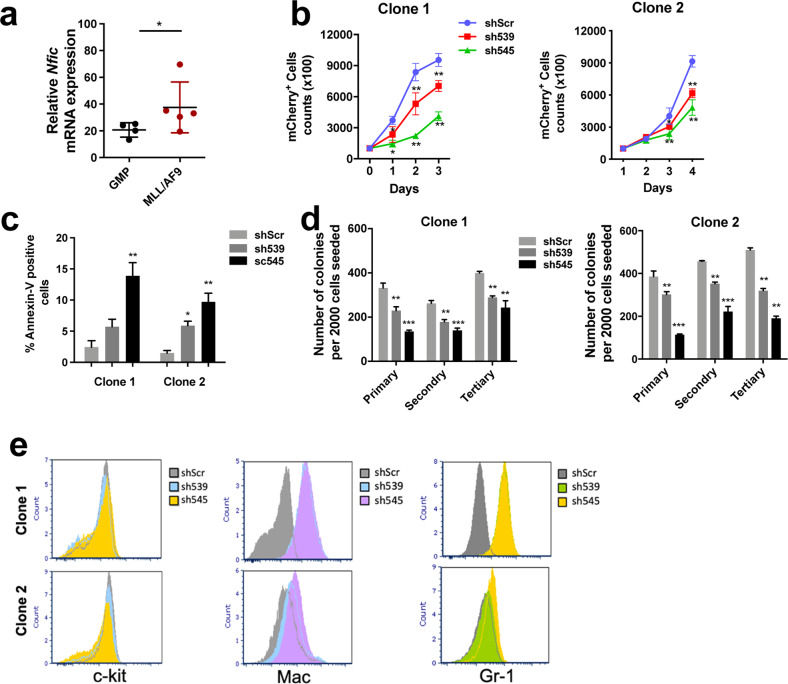
NFIC KD reduces growth, survival and clonogenicity of MLL::AF9 pre-LSCs and induces differentiation and apoptosis. **a** Expression of *Nfic* mRNA in pre-LSCs clones isolated from MLL::AF9 transformed mice (*n* = 5) as compared to their normal counter-part granulo-monocytic myeloid progenitor (GMP) cells (*n* = 4). Each dot represents an individual mouse. Data was analyzed using Mann-Whitney test. **b** Line graph represents growth curve of MLL::AF9 (with GFP) transduced pre-LSCs after *Nfic* knockdown. MLL::AF9 transformed pre-LSCs clones (Clone 1 and 2) form two different mice models were repeat-infected with either *Nfic* shRNAs (sh539 and sh545) or scrambled (shScr) vectors containing m-Cherry. Cells were seeded 48 h post infection. Total cell counts were performed every 24 h for 4 days. Cells were initially gated on GFP for MLL::AF9 and then on m-Cherry. 7-AAD was used to exclude dead cells. Data are mean ± 1 SD with *p < 0.05 and **p < 0.01, analyzed by Dunnett’s multiple comparison test as compared to scramble control. **c** Colony formation assay. The bar graph represents number of colonies per 2000 cells seeded. MLL::AF9 transformed cells were infected with *Nfic* shRNAs or scrambled for 48 h, sorted as GFP^+^/m-Cherry^+^ and seeded in six-well plates containing MethoCult for primary, secondary, and tertiary colony. Colonies were counted and phenotyped after seven days and re-seeded for subsequent colony. Data represented are mean±1 SD (*n* = 4) with *p < 0.05, **p < 0.01 and ***p < 0.001 as analyzed by Tukey’s multiple comparison test. **d** Bar graph represents Annexin-V^+^ cells. MLL::AF9 transformed pre-LSCs were infected with *Nfic* shRNAs or scrambled for 72 h and analyzed for apoptosis assay. 7-AAD was used for necrotic and late-apoptotic cells. Each bar graph shows sum of both Annexin-V^+^ (early apoptotic) and 7-AAD^+^/Annexin-V^+^ (late apoptotic) cell populations. Data represented are mean ± 1 SD (*n* = 4) with *p < 0.05 and **p < 0.01 as analyzed by Dunnett’s multiple comparison test. **e** Flow cytometric histogram plots for c-kit, Mac and Gr-1 expression in tertiary colonies of MLL::AF9 transformed pre-LSCs infected with *Nfic* shRNAs as compared to control (*n* = 4).

To further evaluate the role of NFIC in AML and leukemogenesis we used MLL::AF9 transformed model of leukemia. We found a significant increase in *Nfic* mRNA expression MLL::AF9 transformed pre-LSCs as compared to normal granulo-monocytes progenitor (GMP) cells (Fig. 7a). We next determined the effect of *Nfic* KD on MLL::AF9 expressing pre-leukemic stem cell clones. Initially we screened three mouse-specific *Nfic* shRNAs and scrambled control vectors containing m-Cherry using NIH3T3 cells (Supplementary Fig S7) of which two (sh539 and sh545) were selected for further studies. We next transduced MLL::AF9-GFP pre-LSCs from two different mice (indicated as Clone 1 and Clone 2) with *Nfic* shRNAs and determined their effect on growth and survival. Nfic KD significantly reduced growth of both LSC clones and significantly increased apoptotic cell death (Fig. 7b, c).

To determine the effect of Nfic KD on self-renewal of pre-LSCs, colony assays were performed. A significant reduction in number of colonies of pre-LSCs after Nfic KD in primary, secondary, and tertiary colony formation was observed (Fig. 7d). Phenotypic analysis of these colonies revealed an increase in expression of myeloid differentiation markers Mac1 and Gr-1 in tertiary colonies, in Nfic KD pre-LSCs as compared to pre-LSCs infected with scrambled control (Fig. 7e). However, no change in c-kit expression was observed in any of the colonies suggesting that Nfic KD induces differentiation of MLL::AF9 pre-LSCs. Taken together these data suggest that NFIC may have an important role in growth and survival of MLL::AF9-driven LSCs as well as AML.

## Discussion

Aberrant expression of transcription factors, loss of function mutations and epigenetic regulation have been shown to transform normal hematopoietic stem cells to malignant leukemic cells. Our study demonstrates that transcription factor NFIC is aberrantly expressed in AML and is required for growth and survival of leukemic cells. When overexpressed in normal HSPCs, NFIC promotes survival of these cells under stress conditions, enhance their migration, as well as promoting monocytic differentiation and thereby disrupting normal hematopoiesis.

NFIC belongs to NFI family of proteins all of which have been shown to exhibit both tumor suppressor and oncogenic roles in different cancers [33]. The role of NFI members in AML have been very limited where only the NFIA fusion protein NFIA/CBFA2T3 has been reported in acute erythroid leukemia [34]. In hematopoiesis, NFIA has been shown to play a central role in human erythro-granulopoietic lineage decision by promoting erythroid lineage commitment [35]. On the other hand, NFIX has been shown to promote myeloid differentiation in murine hematopoiesis [36] suggesting a possible redundancy of NFI members in hematopoiesis. In this current study, we found that NFIC promotes human myeloid differentiation particularly with increasing monopoiesis (an effect also supported by scRNA sequencing). Further, a recent study also showed that NFIC overexpression promotes myelopoiesis in murine HSPCs under the direct influence of hepatic leukemic factor (HLF) [37]. However contrary to their findings where overexpression of NFIC alone lead to a decrease in myeloid cells over time we found that NFIC promoted survival of human HSPCs in cytokine-depleted media. A similar effect has been overserved in murine HPSCs where Nfix overexpression supported survival of immature HSPCs, thereby suggesting that these effects can be context and model system dependent.

Single-cell mRNA analysis of human HSPC expressing NFIC, identified 220 genes to be differentially expressed in monocytes compared to control. Most of these genes were enriched in pathways corresponding to cell growth and survival. Among the most significantly upregulated genes where *TYMP* (log_2_FC = 7.11), *HNRNPH1* (log_2_FC = 3.68) and *CDC42* (log_2_FC = 3.68). TYMP encodes for an enzyme thymidine phosphorylase (TP) which is an important component of thymidine catabolism. TP has been shown to confer survival advantage to cancer cells under nutrient deficient conditions [38] and may possibly explain NFIC-HSPCs growth in cytokine-free cultures. On the other hand, HNRNPH1, an RNA binding protein (RBP), has found to be upregulated in AML and is required for AML growth and survival in CRISPR screens for RBPs in AML patient samples [39]. CDC42, a small RhoGTPase is a cell polarity determinant that has been shown to regulate HSC polarity and migration and its increased expression is associated with asymmetric divisions in aging HSC [40]. A recent study has shown that elevated expression and CDC42 GTPase activity is associated with leukemic transformation of normal HSPCs by regulating cell polarity and blocking differentiation stressing the role of CDC42 in leukemogenesis [41].

We also found *S100A12* to be the most downregulated gene (log_2_FC = −10.38) in NFIC overexpressing monocytes. S100A12 is found to be expressed exclusively in leukocytes and has been associated with inflammation-induced activation of myeloid cells. Its exact role in monocytes is not clear but it has been shown that S100A12 is highly expressed in classical monocytes and found to be downregulated in non-classical monocytes [42]. While classical monocytes are the most abundant form of monocytes found in circulation, non-classical monocytes are found to be increased in various disease condition including acute leukemia [10]. Therefore, beyond the scope of the present study it would be interesting to further validate these findings to determine the precise functional role of NFIC in monocytic differentiation and haematopoiesis.

In this study, we also report high nuclear expression of NFIC in AML patient samples as compared to control. Since NFIC belongs to the NFI family of transcription factors (other members being NFIA, NFIB, and NFIX), we analyzed the nuclear protein and mRNA expression of these proteins. *NFIX* was the only family member to have higher mRNA expression compared to control. However, we were not able to detect correspondingly high expression of NFIX protein in the nucleus of AML samples. Interestingly, the samples with high NFIC nuclear levels showed decreased NFIX nuclear localization suggesting a possible redundancy between NFIC and NFIX in AML as well. Although dysregulation of NFIC has been reported in different cancers this study to the best of our knowledge is the first attempt to mechanistically explores its role in AML. We showed that AML cells were dependent on NFIC for their growth and survival and its inhibition induced apoptosis in these cells which was mediated by activation of AIF.

Interestingly, bulk RNA sequencing analysis in THP-1 cells with NFIC KD showed downregulation of MLL target gene, we therefore, analyzed the effect of Nfic KD on cell growth and colony formation using MLL::AF9 pre-LSCs. We observed a reduction in growth and colony formation of these LSCs as well as induced differentiation and apoptosis suggesting that NFIC could be an important transcription factor in MLL::AF9-associated leukemia. Both our single and bulk RNA sequencing analysis showed consensus role of NFIC epigenetic regulation of gene expression in haematopoietic cells, which demands in-depth analysis of NFIC-mediated epigenetic changes in AML cells.

This study shows for the first time that NFIC overexpressing HSPC promotes monocyte development which exhibited distinct molecular characteristics and increased cell survival. We also showed that AML cells depend on NFIC for their growth and survival and that its downregulation not only inhibited growth of AML cells but also impaired normal growth and colony formation in MLL::AF9 pre-LSCs. These findings suggest targeting NFIC can be a potential therapeutic strategy in AML.

## Supplementary information

Supplemental Data

Supplemental Table S2

## Data Availability

The mRNA and scRNA sequencing data sets generated in this study have been deposited in NCBI’s Gene Expression Omnibus. The GEO Series accession number are GSE200456 and GSE196045 respectively.
