# Supplementary material for: Nuclear factor I-C overexpression promotes monocytic development and cell survival in acute myeloid leukemia
Source: Leukemia. 2022 Dec 26;37(2):276–87. doi: 10.1038/s41375-022-01801-z (PMC9898032; doi:10.1038/s41375-022-01801-z)
Supplement: Supplementary file 1 — Supplemental Data [file 41375_2022_1801_MOESM1_ESM.docx]

**Article Title:** Nuclear factor I-C overexpression promotes monocytic development and cell survival in acute myeloid leukemia

**Author Affiliations:** Namrata Rastogi,^1,2*^ Juan Bautista Menendez Gonzalez,^2,3^ Vikas Kumar Srivastava,^1,4^ Bader Alanazi,^1,5,6^ Rehab N. Alanazi,^7^ Owen M. Hughes,^1^ Niamh S. O’Neill,^1^ Amanda F. Gilkes,^1,8^ Neil Ashley,^9^ Sumukh Deshpande,^10^ Robert Andrews,^10^ Adam Mead,^9^ Neil P. Rodrigues,^2^ Steve Knapper,^1,8^ Richard L. Darley,^1^ Alex Tonks^1*^

^1^ Department of Haematology, Division of Cancer & Genetics, School of Medicine, Cardiff University, Cardiff, CF14 4XN, Wales, UK

^2^ European Cancer Stem Cell Research Institute, School of Biosciences, Cardiff University, Cardiff, CF24 4HQ, Wales, UK

^3^ Department of Stem Cell and Regenerative Biology, Harvard Stem Cell Institute, Harvard University, Cambridge, MA

^4^ Division of Infection and Immunity, School of Medicine, Cardiff University, Cardiff, CF14 4XN, UK

^5^ Prince Mohammed Medical City, AlJouf, Saudi Arabia.

^6^ Research Center, King Fahad Medical City, Riyadh, Saudi Arabia

^7^Medical Laboratory Technology Department, College of Applied Medical Sciences, Northern Border University, Arar 91431, Saudi Arabia

^8^ Cardiff Experimental and Cancer Medicine Centre (ECMC), School of Medicine, Cardiff University, Cardiff, CF14 4XN, Wales, UK

^9^ Haematopoietic Stem Cell Biology Laboratory, Medical Research Council Weatherall Institute of Molecular Medicine, University of Oxford, Oxford, OX3 9DS, UK.

^10^ Division of Infection and Immunity, School of Medicine, Cardiff University, Cardiff, CF14 4XN, Wales, UK

***Corresponding authors:** Professor Alex Tonks, Department of Haematology, Division of Cancer & Genetics, School of Medicine, Cardiff University, Wales, CF14 4XN, U.K.

Email: [TonksA@cardiff.ac.uk](mailto:TonksA@cardiff.ac.uk) ; Dr Namrata Rastogi, European Cancer Stem Cell Research Institute, School of Biosciences, Cardiff University, Cardiff CF24 4HQ, Wales, UK. Email: [RastogiN@cardiff.ac.uk](mailto:RastogiN@cardiff.ac.uk).

**Supplemental Materials and Methods**

**Supplementary Tables**

**Supplementary Figures**

**Supplemental Materials and Methods**

**Cell culture**

AML cell lines were obtained from ATCC (Middlesex, U.K.) or ECACC (Salisbury, U.K.) and cultured under recommended culture conditions. Briefly, HL60, THP-1, HEL, NB4, and U937 were cultured in RPMI and OCI-AML2 was cultured in IMDM, growth media supplemented with +10% (*v/v*) fetal bovine serum and 1% (*v/v*) penicillin / streptomycin. Cells were maintained in a humidified incubator with 5% CO_2_ in air and used within passage twenty for all experiments. Short tandem repeat analysis was performed to confirm the genetic integrity of the cell lines. Mycoplasma contamination was monitored through MycoAlert Detection kit (Sigma, Dorset, U.K).

**Plasmids**

NFIC overexpression studies were performed using lentiviral expression vector pLV-EFIA-NFIC-EGFP-T2A-Puro containing *NFIC* transcript (NM_001245002.2) and purchased from Vector Builder (Guangzhou, China). For knock down studies, NFIC or AIF short-hairpin RNAs were selected from Broad institute (see below). shRNA and control vectors were designed on pLV-U6-[shNFIC/Scrambled RNA]-EGFP-T2A-Puro or U6-[shAIF/Scrambled RNA]-mCherry-T2A-Puro plasmid backbone and purchased from Vector Builder.

| Vector | TRC | Sequence 5’ to 3’ | Target | Selectable Marker |
| --- | --- | --- | --- | --- |
| shScr | Scrambled shRNA | Scrambled (GFP) | Human | GFP |
| sh473 | TRCN0000014712 | CCTCCGCTCTGCATTTCCCTA | Human | GFP |
| sh488 | TRCN0000014708 | CCACCCAAGCAAGAAGACAAA | Human | GFP |
| sh494 | TRCN0000277970 | GATGGACAAGTCACCATTCAA | Human | GFP |
| sh481 | TRCN0000014711 | CCCGGTGAAGAAGACAGAGAT | Human | GFP |
| sh501 | TRCN0000014710 | GCGGCACAAATCGGGCTCGAT | Human | GFP |
| shScr2 | Scrambled shRNA | Scrambled (mCherry) | Human | mCherry |
| Sh584 | TRCN0000229861 | CTGCATGCTTCTACGATATAA | Human | mCherry |
| Sh601 | TRCN0000229860 | TTTGGTGGCTTCCGGGTAAAT | Human | mCherry |
| Sh605 | TRCN0000218045 | AGATTTCACGGGAAGTCAAAT | Human | mCherry |
| shScr | Scrambled shRNA | Scrambled (GFP) | Mouse | GFP |
| sh539 | TRCN0000310992 | TGTGTGCAGCCGCACCATATT | Mouse | GFP |
| sh545 | TRCN0000310995 | GCAACTGGACGGAAGACATAG | Mouse | GFP |
| sh550 | TRCN0000310997 | ACCTGTACCTGGCCTACTTTG | Mouse | GFP |

**Immunofluorescence**

For intracellular protein analysis, cells were harvested four days post infection, washed with PBS and fixed with 4% (*v/v*) paraformaldehyde for ten min. Fixed cells were washed with PBS twice and permeabilized with 0.3% (*v/v*) Triton-X100 solution in PBS for 5 min. Following incubation, cells were washed with PBS and blocked for 30 min with 5% (*wv/v*) Bovine Serum Albumin (BSA). Following blocking, cells were pelleted and incubated with primary antibodies NFIC (Abnova) (1:100) and Anti-Mouse-IgG (Sigma) (1:100) over night at 4^o^C. Cells were washed with PBS+1% (*v/v*) BSA and incubated with secondary antibody F(ab’)2-Alexa Fluor® 647 secondary (CST) (1:1000) for 1h at RT. Cells were washed and resuspended in PBS+1% (*v/v*) BSA and analyzed by flow cytometry.

**Flow cytometry data analysis.**

For differentiation assay, transduced human CD34^+^ HSPCs were incubated with CD36-Biotin, CD13-APC, CD34-PE, CD11b-PE, CD14-PE, or CD15-PE at 4^o^C for 30 min. Biotin labelled antibody was detected following a secondary incubation step with Streptavidin-PerCP-5.5. Cells were washed and resuspended in PBS + 1% BSA and flow cytometric data acquired using flow cytometry. Cells were gated on GFP and then analyzed for cell surface markers; classified as monocytes (CD15^lo^CD14^hi^CD36^lo­^), granulocytes (CD15^hi^CD14^lo^CD36^lo^) and erythrocytes (CD15^-^CD14^-^ CD36^hi^).

For cell surface protein expression of MLL/AF9 pre-leukemic clones, cells were washed with PBS+2%FBS (*v/v*) and incubated with c-Kit-APC, Mac1-APC, Gr-PE-Cy7 (Biolegend) for 30 min at 4^o^C. Cells were subsequently washed with PBS+2%FBS (*v/v*) and resuspended in PBS+2%FBS (*v/v*) containing DAPI (10 μg/mL) and analyzed by flow cytometry. For data analysis cells were gated on GFP and m-Cherry. GFP^+^mCherry^+^ cells were analyzed for c-kit as well as Mac1 and Gr1 expression for myeloid differentiation.

**Single cell nuclei preparation, cDNA synthesis and library preparation**

Human CD34^+^ HSPCs were FACsorted following four days of transduction with NFIC or GFP only vector for GFP positive live cells using 7-actinomycin-D (7AAD). Cells were washed and resuspended in PBS with 0.04% BSA (*v/v)* at a density of 600-700 cells /μL as per the recommended protocol for cell preparation for 10x Genomics (10× Genomics, Pleasanton, CA) single cell RNA sequencing protocol. Gel-Beads in emulsion (GEM) were generated using Chromium Next GEM Single Cell 3ʹGEM Kit v3.1 (10x Genomics). 10,000 cells per sample were mixed with freshly prepared master mix from the reagents provided and loaded on Chromium Next GEM Chip G along with barcoded gel beads (containing 10× cell barcodes, unique molecular identifiers (UMI) and poly(dT) sequences) and partitioning oil on their respective lanes. The chip was then loaded onto a Chromium Controller (10× Genomics) for single-cell GEM generation and barcoding at single cells resolution. Barcoded full length cDNAs generated from polyadenylated RNA were recovered from pooled fractions for one sample and PCR amplified to generate sufficient cDNA mass for library construction. Amplified cDNA was quantified using Qubit 4.0 Fluorometer (Thermo fisher Scientific). cDNA libraries were constructed using Chromium Next GEM Single Cell 3ʹ Library Kit v3.1 (10x Genomics). Amplified cDNA was fragmented and mixed with sample indexes and adapters for library generation. The size and profiles of the sequencing libraries generated were examined by Agilent Bioanalyzer 2100 using High Sensitivity DNA chip (Agilent).

**Single cell RNA sequencing and analysis**

Single cell cDNA libraries generated were sequenced using Illumina NextSeq 500 system using the NextSeq 500/550 High Output v2 Kit (Illumina, San Diego, CA). Sequencing was performed to obtain 400 million reads covering an average of 50,000/ cells. Sequencing data was analysed using 10x Genomics Cell Ranger Pipeline v4.0 and R studio. Raw sequencing base call (.bcl) files from Illumina was demultiplexed using cellranger mkfastq to generate FASTQ files for further analysis in R studio.

Data from 10X Genomics was imported into CellRanger software v.3.1.0. Raw sequencing BCL files from control and overexpression samples were demultiplexed into paired-end FASTQ files using “cellranger mkfastq”. “cellranger count” was then used to perform read alignment, UMI counting and secondary analysis separately for each sample. Human reference genome GRCh38 was used as reference for generating counts from FASTQ files. “cell ranger aggr” was used to aggregate the output from “cellranger count”, normalization and secondary analysis was performed on the combined dataset. Data was further analysed using Seurat v.3.1.5. [1] Cells were filtered having unique feature counts less than 2,500 and greater than 200 and cells having mitochondrial gene counts less than 5%. Filtered cells with unique molecular identifier counts were log normalized to derive cells with normalized feature counts. Variable genes were selected and identified based on the outliers on a “mean variability plot”. Variance Stabilizing Transformation (VST) method was to select the top variable genes [2]. Data was scaled and dimensionality was reduced by principal component analysis, performed using the variable genes. Out of 30 significant PCs for each sample, 11 PCs were selected with the Elbow Plot function. RunTsne and RunUMAP were applied for visualization of the selected PCs to the cells in two dimensions. Clusters were identified with FindAllMarkers function was used for identifying cluster markers with the parameter “only.pos” set to TRUE, “min.pct” to 0.25 and “logfc.threshold” set to 0.25. Markers genes were identified having adjusted p-value less than equal to 0.05 using the non-parametric Wilcoxon rank-sum test [3].

FASTQ files were also analysed using Partek Flow single cell analysis module (Version: 6.0.17.1206) using the 10x Genomics Chromium™ Single Cell 3' v2 prep kit. hg38_ensembl_release90_v2 was used for feature/gene annotation. Similar filtering criteria was used as above for QA/QC analysis. PCA and Group based as well as ‘K’ means Clustering resulted in cell clusters where each cluster was identified by the unique gene expression pattern as described above analysis. Differential gene expression analysis for monocyte cluster between NFIC overexpressed and control was performed using Partek Flow’s in-build algorithm (Supplemental Table S2).

**Bulk mRNA sequencing**

THP-1 cells transduced with NFIC shRNA or GFP only vector were FACsorted for GFP four days following transduction, in ice cold PBS containing 1% (*v/v*) FBS and RNAse inhibitor. Sorted cells were centrifuged for 280 × g for 10 min at 4^o^C and lysed in RLT Plus buffer. Total RNA was isolated using All Prep DNA/RNA kit (Qiagen GmBH, Hilden, Germany) according to manufacturer’s protocol. Quality of RNA was determined using Bioanalyzer 2100 (Agilent) and all samples with RNA integration number (RIN) of >9 was used for sequencing analysis. RNA-seq library was generated using NEB Next® Ultra RNA Library Prep Kit (Illumina) according to manufacturer’s protocol. Libraries were analysed by pair-end sequencing using Novaseq 6000 S4.

**Bulk mRNA sequencing analysis**

Paired-end reads from Illumina sequencing were trimmed with Trim Galore

(<https://www.bioinformatics.babraham.ac.uk/projects/trim_galore/>) and assessed for

quality using FastQC, (<https://www.bioinformatics.babraham.ac.uk/projects/fastqc/>)

using default parameters. Reads were mapped to the human GRCh38 reference genome using STAR [4] and counts were assigned to transcripts using featureCounts [5] with the GRCh38.96 HEnsembl gene build GTF. Both the reference genome and GTF were downloaded from the Ensembl FTP site [<http://www.ensembl.org/info/data/ftp/index.html/>]. Differential gene expression analysis was performed using DESeq2 package [6] Significance was calculated as ‘*p*’ and adj. ‘*p*’ (using Benjamini-Hochberg correction) values. Geno ontology and pathway analysis were performed using Gene Set Enrichment Analysis (GSEA) software (version 4.1.0), Ingenuity Pathway Analysis (IPA) (Qiagen), and MetaScape [7].

**Total, nuclear, and cytosolic protein extraction**

Cytosolic fraction of cells was isolated using Nuclear-Cytosolic extraction kit (Biovision) according to manufacturer’s instructions. For nuclear protein extraction, pellet obtained post cytosol extraction was snap frozen and thawed in three freeze-thaw cycles in liquid N_2_. Pellet was then incubated with 50U Benzonase (Merck-Millipore, UK) for 30 min on ice. Following incubation, pellet was lysed using triethylammonium bicarbonate (TEAB) buffer (0.5M TEAB, 0.05% (*v/v*) SDS, Protease Inhibitor cocktail and Phosphatase inhibitor cocktail) (Sigma-Aldrich, UK) for 30 min on ice with vortexing every 10 min. The lysate was centrifuged at 10,000×g at 4^o^C for 10 min and supernatant containing the nuclear proteins was collected in a fresh-chilled tube.

Total protein was isolated using RIPA buffer (Sigma-Aldrich) where cells were harvested and washed with ice cold PBS. Cell membrane was lysed with three freeze-thaw using liquid N_2._ Pellet was lysed with RIPA buffer containing Protease and Phosphatase inhibitor cocktail on ice for 30 min with vortexing every 10 min. Lysate was centrifuged at 10,000×g at 4^o^C for 20 min and supernatant was collected. Proteins were quantified using standard Bradford assay as described previously [8].

**Supplementary Figures**

**b**


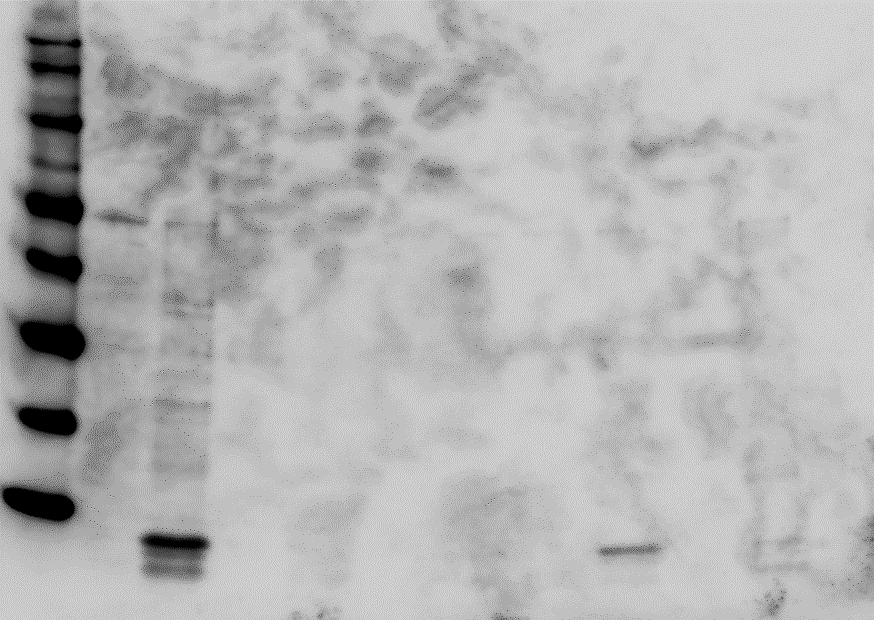

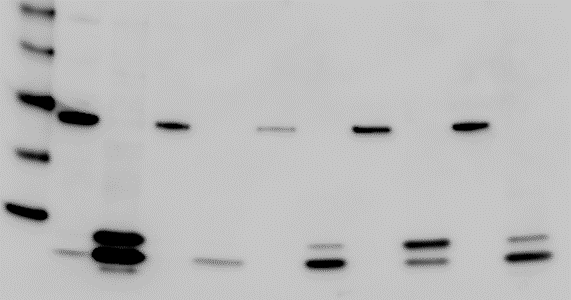

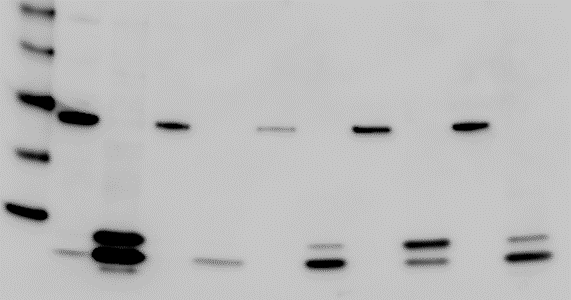


(C) (N)

**BM1**

(C) (N)

**BM2**

(C) (N)

**CD34^+^**

**GAPDH**

**H1**

**NFIC**

**a**


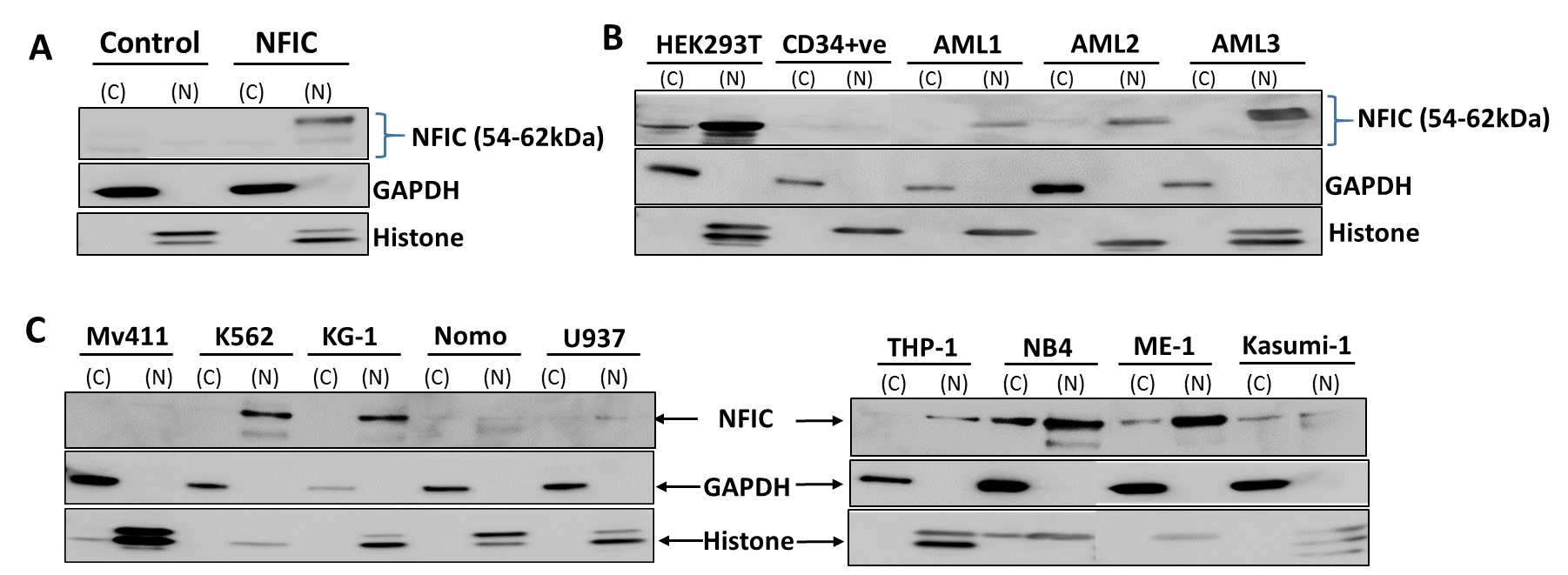


**HEK-293T**

**GAPDH**

**H1**

**NFIC**

**Supplemental Fig. S1. Validation of detection of NFIC protein in HEK cells and normal human CD34^+^ HSPC and human bone marrow.** Immunoblots showing expression of NFIC in cytosolic (C) and nuclear (N) protein extracts. **a** HEK-293T cells were transfected with NFIC overexpression vector (NFIC) or control (expressing GFP alone). **b** Expression of NFIC in CD34^+^ derived HSPC and two independent normal human bone marrow (BM1 and BM2) samples (total mononuclear cells). GAPDH and Histone 1 (H1) were used as loading controls for cytosolic and nuclear lysate respectively.


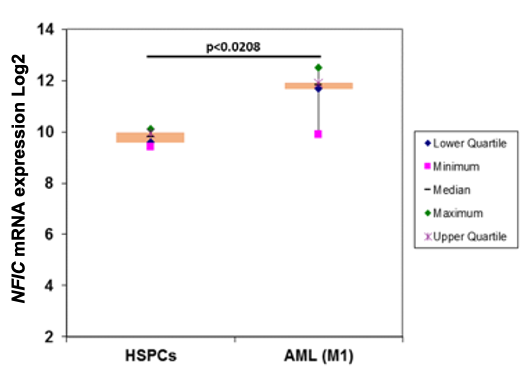


**b**

**a**

**Supplemental Fig. S2.** ***NFIC* mRNA expression is increased in AML compared to normal human hematopoietic cells**. **a** Box-Whiskers plot of *NFIC* mRNA expression (log_2_) in normal CD34^+^ human stem and progenitor cells (HSPCs) and AML patient samples of FAB M1 sub-type (n=3 HSPC and n=15 AML). Significant difference was analyzed by Mann-Whitney U, *p<0.05. **b** *NFIC* mRNA expression in different AML subtypes; Acute promyelocytic leukemia with t(15;17) (PML::RARA), Inversion16 with t(16;16) (CBFB::MYH11), RUNX1::RUNX1T1 with t(8;21), Mixed Lineage Leukemia (MLL) with t(11q23) and AML with Complex Karyotype. mRNA expression within different molecular subsets of AML as compared too normal hematopoietic stem cells (HSCs) using data sets GSE42519 (refs [9,10]) (for AML) and GSE13159 (ref [11]) (for normal blood cells). Data was analyzed using Bloodspot software [12]. Statistical analysis was performed using Tukey’s test to analyze the level of statistical significance of AML subtypes compared to normal HSCs where **p<0.01, ***p<0.001, ****p<0.0001


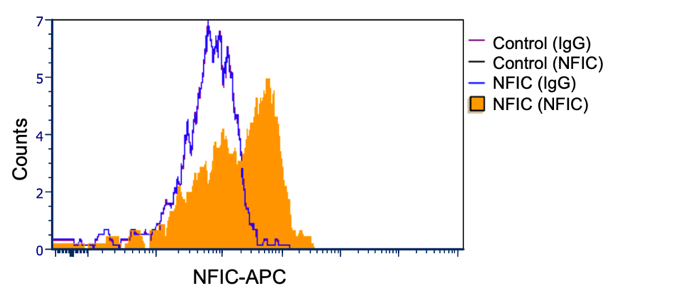
**
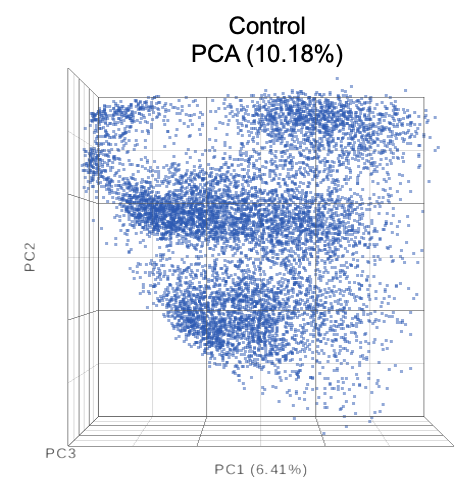

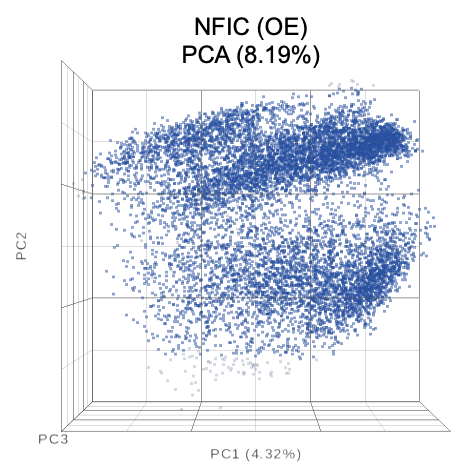
**

**a**

**c**

**b**

**
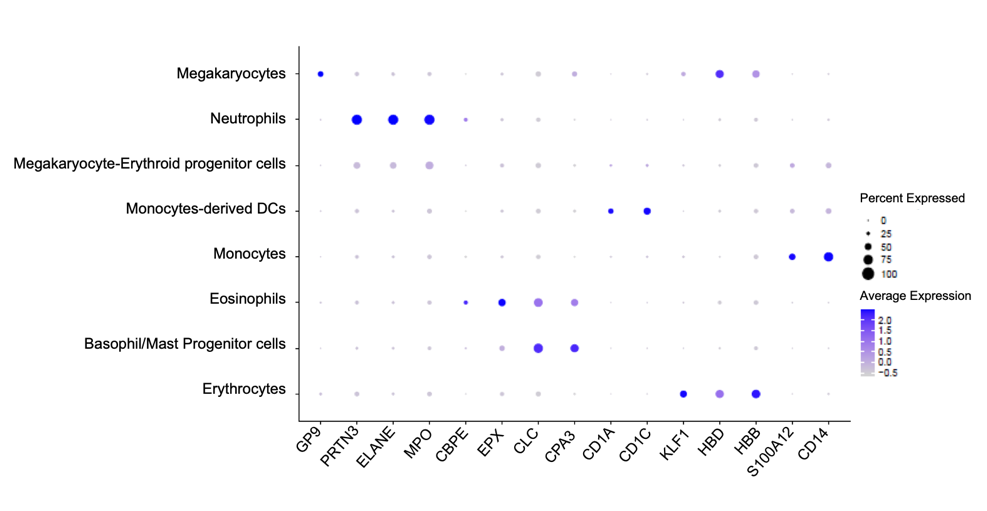

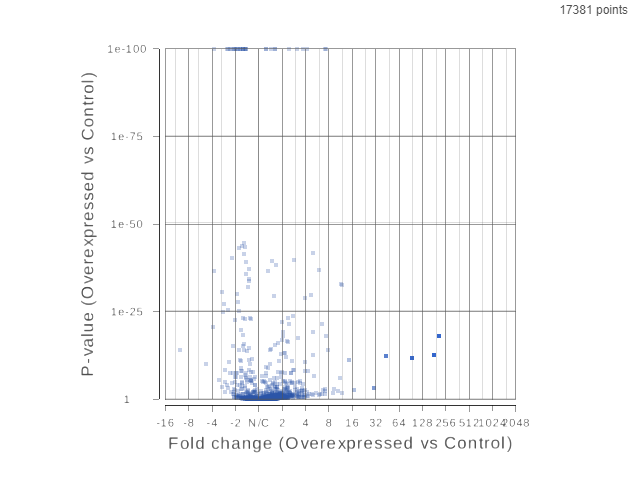
**

**d**

**Supplemental Fig. S3. Single cell RNA sequencing of NFIC overexpressing HSPC.** **a** Flow cytometric histogram showing expression of NFIC protein in normal CD34^+^ HSPC. CD34^+^ enriched cells from normal human cord blood were infected with GFP only (Control) or NFIC overexpression (NFIC) vector. Four days post infection cells were fixed, permeabilized and stained with anti-NFIC or isotype control (IgG) antibodies labelled with Alexa-Fluor 647. **b** To reduce dimensionality of the scRNA seq data, Principal Component Analysis (PCA) was applied prior to clustering different cell populations though K-means clustering. PCA plots for single cell mRNA sequencing of Control and NFIC overexpressing (OE) blood cells. Normalized gene expression was subjected to PCA to reduce dimensionality before clustering the cells using Partek Flow software. **c** Dot plot representing expression intensities of highly expressed gene signatures based on PCA across different cellular clusters identified after K-means clustering. Each dot represents an individual protein, the colour of the dot indicates its average expression in any given cluster whereas its size indicates percent cells expressing that protein within a cluster. Based on the cellular and functional identities, these clusters were classified according to their terminal differentiation as monocytes, erythrocytes, dendritic cells (DC), megakaryocytes, neutrophils, megakaryocyte-erythroid progenitor cells, basophil/mast progenitor cells and eosinophils. **d** Genes differentially expressed (DE) within the monocytes cell cluster was subjected to volcano plot with X-axis representing average Fold change and Y-axis represents *p* value.


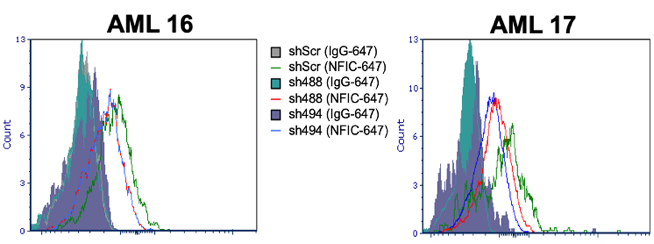

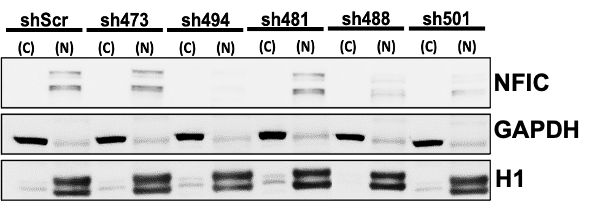


**b**

**a**

**c**

**
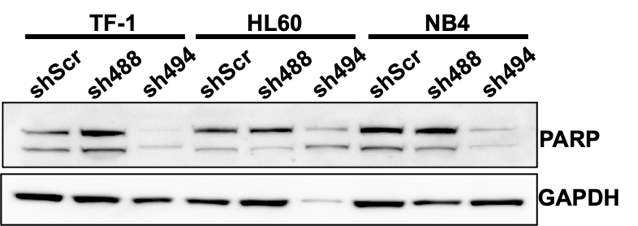

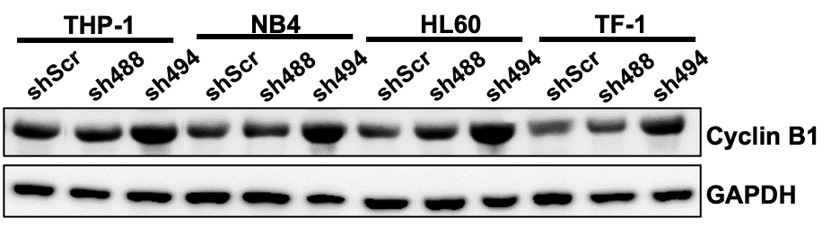
**

**d**

**e**

**Supplemental Fig.S4.** **NFIC knockdown induces cell cycle arrest in AML cells. a** Western blot showing expression of NFIC following knockdown (KD) in HL60 cells. HL60 cells were infected with either of five different shRNAs or scrambled control (shScr). Cells were selected for four days with Puromycin (1μ/mL). Cytosolic (C) and nuclear (N) lysates from puromycin selected cultures were isolated and immunoblotted for NFIC protein expression (n=1). GAPDH and Histone H1 were used as loading controls for cytosolic and nuclear lysates respectively. **b** Flow cytometric histogram plots showing KD efficiency of two selected shRNAs (sh488 and sh494) in AML patient samples (AML 16 and AML 17) as compared to scramble control. **c** Bar graph showing percentage of cells in different cell cycle phases. AML cell lines were infected with either sh488 and sh494 or shScr. Four days post-infection cells were fixed in 70% *v/v* ethanol for 30 min on ice, washed and stained with propidium iodide (PI) and analyzed by flow cytometry. GFP^+^ cells were analyzed for percentage of cells in different cell cycle phases. Data represents mean ± 1SD (n=3). **d and e** Immunoblots showing expression of Cyclin B1 and Parp respectively. Infected AML cell lines were selected with puromycin for four days. Selected cells were incubated further for three days and analyzed by western blot. GAPDH was used as an endogenous control (n=2).

**a**


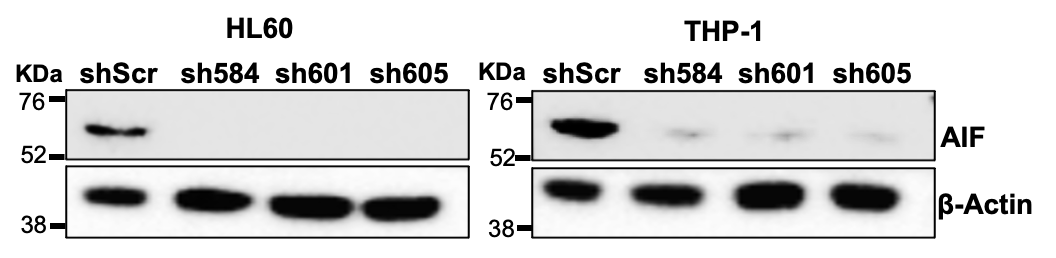


**b**


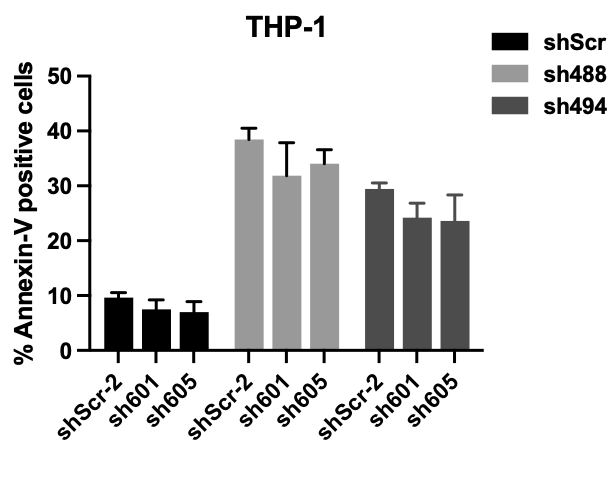

**Supplemental Fig. S5. Effect of AIF knockdown on NFIC depleted AML cells. a** Immunoblot showing expression of AIF protein following knockdown in HL60 and THP-1 cells. Both cell lines were infected with three different shRNAs or scrambled control. Infected cells were selected for four days with Puromycin (1μg/ml). Total protein lysate was extracted and western blotting was performed for AIF protein expression. β-actin was used as endogenous controls. **b** Bar graphs showing precent Annexin-V positive cells. HL60 and THP-1 cells were seeded and infected with either shRNA for AIF (m-Cherry^+^) alone, co-infected with shRNAs for NFIC (GFP^+^) and AIF (m-Cherry^+^) or scrambled controls as indicated. Four days post-infection cells were harvested, washed and stained with Annexin-V-APC and analyzed through flow cytometry. Data represents mean ± 1SD (n=3).


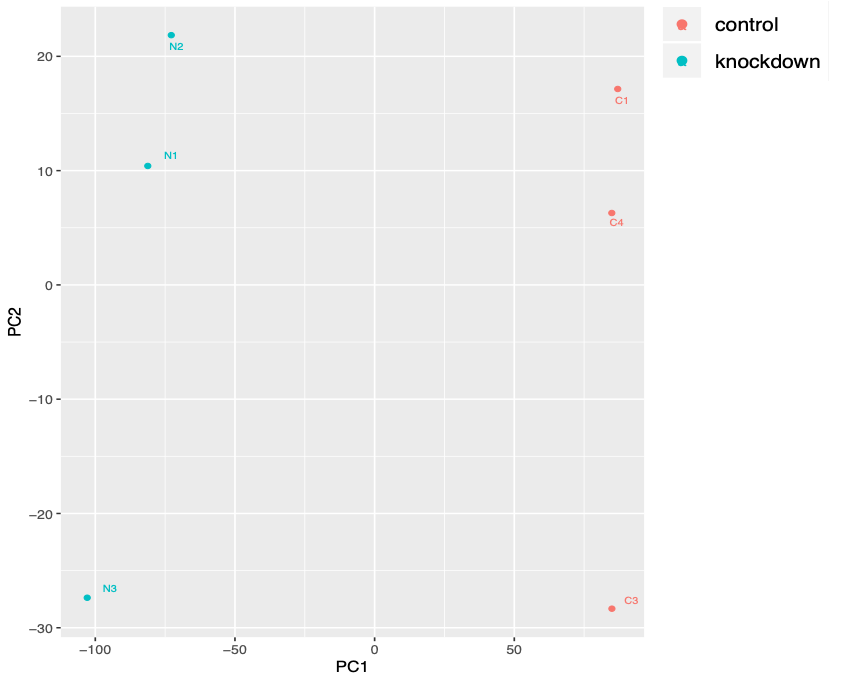


**a**

**b**


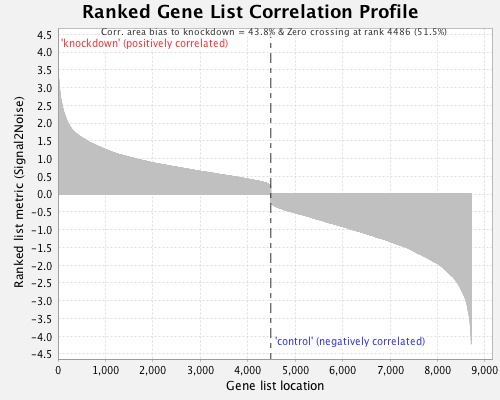


**
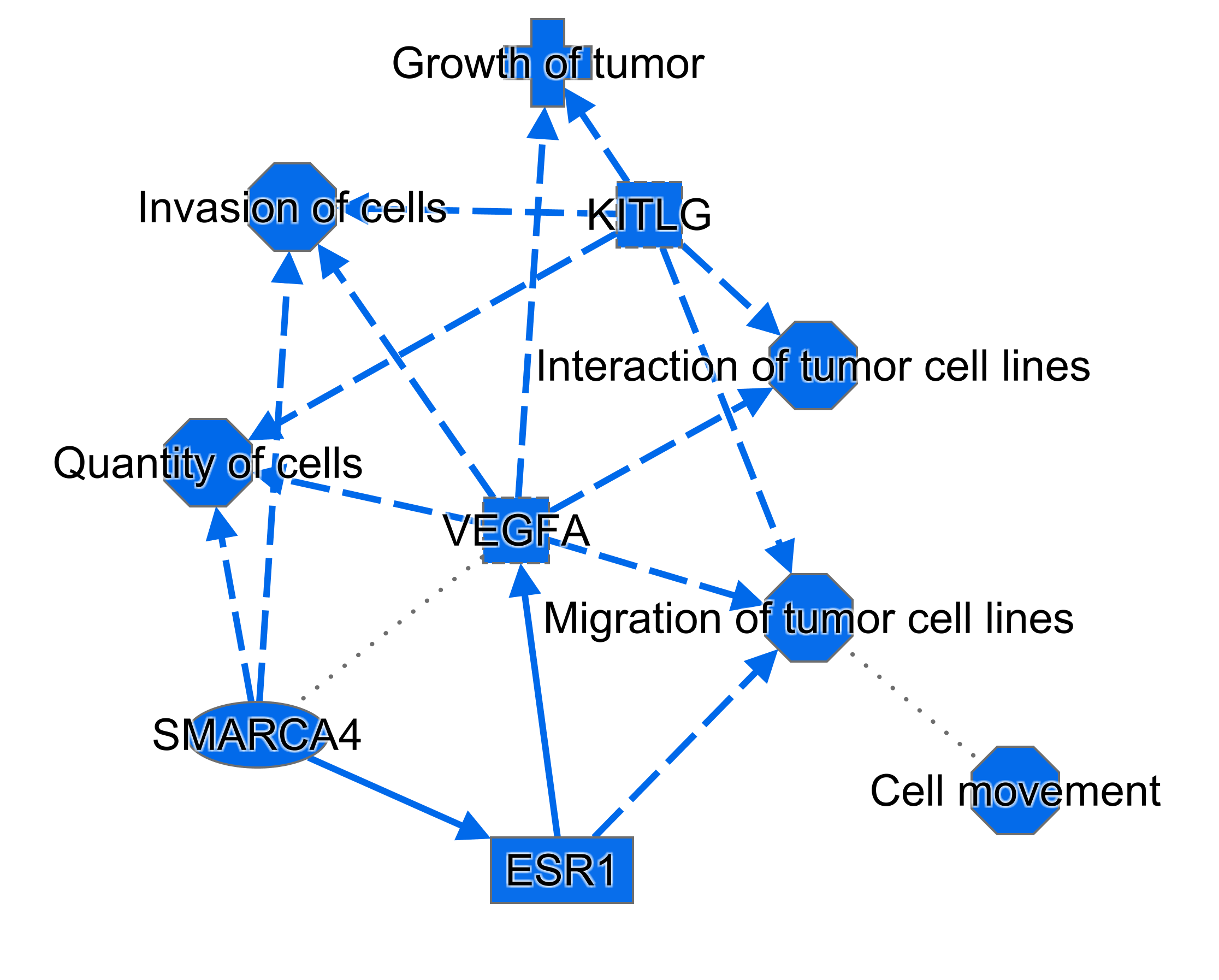
**

**
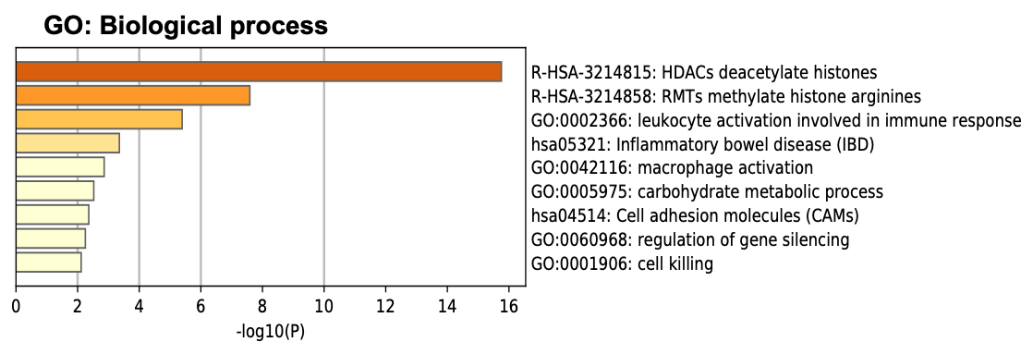
**

**c**

**d**

**Supplemental Fig. S6. mRNA sequencing analysis of NFIC KD THP-1 cells a** PCA plot showing special orientation gene expression data. Processed FPKM values from gene counts were plotted against two principal components (PC1 and PC2). **b** Ranked gene list correlation profile graph. DE genes from THP-1 knockdown vs scramble control were ranked according to their order of expression for GSEA pathway enrichment analysis based on the normalized gene counts. **c** Graphical summary of IPA analysis. Representative graphical summary of Pathway analysis of gene differentially expressed genes in NFIC knockdown as compared to scrambled control was done using IPA. Diagram represents the graphical summary of the RNA seq data showing interactions between major molecular functions, growth of tumor, migration of tumor cell lines, cell movement, invasion, and upstream regulator genes VEGFA, SMARC4, KTLG and ESR1. The direct and indirect interactions are represented by solid lines and dashed lines respectively. d Graph representing Gene Ontology (GO) analysis using Metascape for all the genes significantly upregulated (FDR<0.05, log2 Fold change >1.5) showing enrichment of pathways involved in Histone deacetylation, cell adhesion, gene silencing and cell killing.


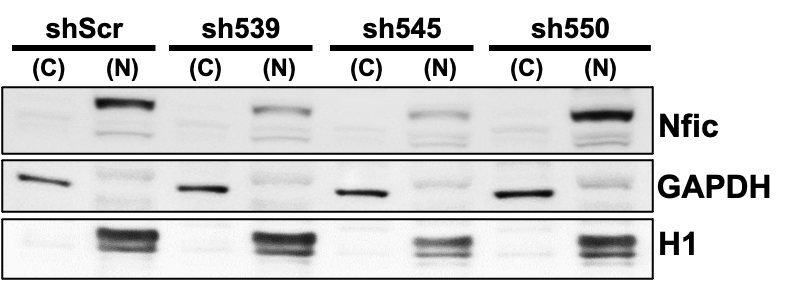


**Supplemental Fig. S7.** Immunoblot showing expression of Nfic following knockdown in NIH3T3 cells. NIH3T3 cells were infected with three different shRNAs or scrambled control. Infected cells were selected for four days with Puromycin (1μg/ml). Cytosolic (C) and nuclear (N) protein extraction western blotting was performed for Nfic protein expression. GAPDH and histone H1 were used as endogenous controls for cytosolic and nuclear lysates respectively (n=1).

**Supplemental Tables**

**Supplemental Table S1. Clinical characteristics of samples used in the study.**

| Sample  number | Sample  type | Age  (y) | Sex | FAB Sub-type | Cytogenetics | Mutations tested  (NPM1/FLT3-TKD/FLT3-ITD) | Diagnosis | WBC *(x10^9^/L)* | Treatment |
| --- | --- | --- | --- | --- | --- | --- | --- | --- | --- |
| *CD34* | CB-CD34 | n/a | n/a | N/A | n/a | n/a | n/a | n/a | n/a |
| *BM1* | BMMNCs | n/a | n/a | N/A | n/a | n/a | n/a | n/a | n/a |
| *BM2* | BMMNCs | n/a | n/a | N/A | n/a | n/a | n/a | n/a | n/a |
| *AML1* | AML (PB) | 63 | F | M1 | Failed | WT / WT / WT | De Novo | 46 | Daunorubicin 35 + cytarabine 200 |
| *AML2* | AML (PB) | 68 | F | M1 | 46,XX [20] | Type D / WT / ITD | De Novo | 114 | DA  (3 courses) |
| *AML3* | AML (BM) | 77 | M | M1 | 46,XY [20] | Type A / WT / WT | De Novo | 130 | LDAC  +ATO |
| *AML4* | AML (PB) | 49 | M | M1 | NK | Type A / WT / ITD | De Novo | 153 | DA60 + GO |
| *AML5* | AML (PB) | 17 | M | M1 | 46,XY [20] | WT / WT / WT | De Novo | 294 | FLAG-IDA+GO |
| *AML6* | AML (PB) | 48 | F | M1 | 46,XX,t(9;11)  (p22;q23) [10] | WT / WT / WT | De Novo | 46 | ADE |
| *AML7* | AML (BM) | 55 | F | M1 | 46,XX [20] | Type B / WT / WT | De Novo | 77 | DA60 |
| *AML8* | AML (PB) | 54 | F | M1 | 46,XX [15] | Type A / WT / ITD | De Novo | 39 | ADE+GO |
| *AML9* | AML (PB) | 55 | M | M1 | 46,XY [5] | Type A / D835Y / ITD | De Novo | 254 | Daunorubicin 35 + cytarabine 200 |
| *AML10* | AML (PB) | 17 | M | M1 | 46 XY[20] | WT/ NA / WT | De Novo | 294 |  |
| *AML11* | AML (PB) | 45 | F | M1 | 46 XX [20] | Type A / WT / 69bp ITD 33% | De Novo | 70.6 | DA Standard dose (60mg) |
| *AML12* | AML (PB) | 63 | F | UNKNOWN | 46,X,?idic(X)(q11),add(7)(q22),-8,der(?10)t(7;10)(q22;q2?4),+mar[10]/46,XX[1] | n/d | De Novo | 17.5 | n/d |
| *AML13* | AML (PB) | 24 | M | M4 | 46,XY,inv(16)(p13q22)[16].ish inv(16)(p13)(MYH11+,CBFB+)(q22)(CBFB+,MYH11+)[5] | WT /WT /WT | De Novo | 255.8 | n/d |
| *AML14* | AML (PB) | 67 | M | M1 | NK | WT / WT / 51bp ITD 43% | Secondary | 50.7 | n/d |
| *AML15* | AML (PB) | 57 | F | M1 | 46 XX | n/d | De Novo | 145.5 |  |
| *AML16* | AML (PB) |  | F | M1 | 46,XX,inv(16)(p13q22)[18]/  46,XX[2] | WT / WT / WT | Secondary | 70 | DA Standard dose (60mg) |
| *AML17* | AML (PB) | 55 | F | M2 | 47,XY,+8[1]/46~47,XY,+8,-13,-13,del(13)(q?12q?22),add(17)(p11.2),+3mar[cp10]/46,XY[1] | WT / WT / WT | De Novo | 15.8 | DA Standard dose (60mg) |
| *AML18* | AML (PB) | 62 | F | M5 | 46,XX[20] | Type A / WT/ WT | De Novo | 44.7 | DA Standard dose (60mg) |
| AML 19 | AML(PB) | 54 | M | M2 | N/A | WT/ WT/ WT | De Novo | 47.8 | Daunorubicin 35 + cytarabine 200 |
| *AML20* | AML(PB) | 61 | F | M1 | N/A | n/d | De Novo | 100.2 | n/d |
| *AML21* | AML(BM) | 64 | M | M3 | 46,XY | Type A / WT/ WT | De Novo | 76 | DA Standard dose (60mg) |
| *AML22* | AML(PB) | 68 | M | M2 | 46,XY[20] | Type D / WT / ITD | De Novo | 98 | n/d |

FAB, French American British classification system; WT, Wild Type; NPM1, Nucleophosmin 1; FLT3-TKD, Fms-Like Tyrosine Kinase 3- Tyrosine Kinase Domain; FLT3-ITD, Fms-Like Tyrosine Kinase 3-Internal tandem duplication; ATO, Arsenic Trioxide; CB, Cord Blood; PB, Peripheral Blood; BM, Bone Marrow; BMMNCs, Bone Marrow mononuclear cells; DA, Daunorubicin/Cytarabine; FLAG-Ida, Fludarabine, Cytarabine, GMCSF-Idarubicin; ADE, Cytarabine/ Daunorubicin/ Etoposide; GO, Myelotarg; N/A, Not Applicable; NK, Normal Karyotype; n/d, No data; WBC, White Blood Cell Count; LD, Low Dose.

**Supplemental Table S2:** List of differentially expressed genes in cells of monocytic lineage overexpressing NFIC as compared to control vector generated using Partek Flow software. (Attached as separate excel sheet with details)

**Supplemental Table S3. Differentially expressed genes from the cell survival network analysis.**

Network analysis map was constructed using IPA tool. The genes enlisted in the cell growth and survival pathway from the mechanistic network analysis were selected to create the map using the IPA’s Build tool and Grow tool. The RNAseq data was overlayed to the network for Fold change expression values and gene counts of each gene as well as disease and molecular functions to display the interconnected functional nodes within these genes.

| Gene Symbol | Fold Change | p-value | False Discovery Rate (q-value) | Gene counts |
| --- | --- | --- | --- | --- |
| B2M | 1.6 | 5.91163E-82 | 9.34092E-79 | 6847 |
| CCL2 | 2.3 | 3.915E-05 | 0.00352573 | 113 |
| CD44 | 1.8 | 1.96226E-08 | 2.60351E-06 | 424 |
| CD74 | 1.7 | 4.72604E-39 | 1.91031E-36 | 2886 |
| CDC42 | 3.7 | 1.60419E-88 | 3.4853E-85 | 1402 |
| CTNNB1 | 7.8 | 1.23064E-14 | 2.20512E-12 | 140 |
| DUSP1 | -2.0 | 1.092E-05 | 0.00105537 | 174 |
| FOS | -4.8 | 6.79949E-11 | 1.01881E-08 | 121 |
| GPX1 | 1.6 | 5.564E-61 | 4.83539E-58 | 5034 |
| HLA-A | 2.6 | 3.78897E-08 | 4.80701E-06 | 165 |
| IL1B | -1.6 | 3.84458E-19 | 8.56699E-17 | 1473 |
| ITGB2 | 1.6 | 0.00013569 | 0.011560927 | 264 |
| JUN | -3.0 | 0.000442123 | 0.035576548 | 54 |
| JUND | 2.5 | 3.29225E-55 | 2.38428E-52 | 1458 |
| LDHA | 1.6 | 0.00043996 | 0.035567209 | 216 |
| LMNA | 4.9 | 1.74721E-42 | 8.20762E-40 | 524 |
| MAP3K8 | 3.3 | 2.56227E-05 | 0.002331665 | 69 |
| NEAT1 | 2.4 | 4.54022E-17 | 9.28396E-15 | 455 |
| PKM | 1.5 | 2.63487E-05 | 0.002385247 | 398 |
| PLSCR1 | 2.5 | 4.88469E-06 | 0.00051455 | 118 |
| PPIA | -1.6 | 9.54435E-06 | 0.000942559 | 343 |
| PPP1CB | 6.6 | 3.738E-22 | 8.90002E-20 | 230 |
| RPL27 | -1.8 | 9.42861E-15 | 1.74339E-12 | 747 |
| RPL35A | -1.7 | 2.80252E-46 | 1.52221E-43 | 3152 |
| RPL38 | -2.5 | 3.51506E-26 | 1.00156E-23 | 655 |
| SAT1 | 2.1 | 7.21576E-14 | 1.25417E-11 | 451 |
| SDCBP | 2.7 | 1.60484E-11 | 2.46847E-09 | 233 |
| TIMP1 | 2.1 | 5.21269E-20 | 1.19213E-17 | 737 |
| TPT1 | -1.6 | 2.52108E-71 | 2.73868E-68 | 5839 |
| TYMP | 7.1 | 5.7044E-97 | 1.65247E-93 | 1052 |
| VCAN | -2.8 | 5.73949E-28 | 1.66263E-25 | 581 |
| WTAP | 11.3 | 1.22884E-33 | 4.18793E-31 | 333 |

**Fold Change**: Log_2_ Fold change of expression in NFIC overexpression vs Control.

**References:**

1 Butler, A., Hoffman, P., Smibert, P., Papalexi, E. & Satija, R. Integrating single-cell transcriptomic data across different conditions, technologies, and species. *Nat Biotechnol* **36**, 411-420 (2018). <https://doi.org:10.1038/nbt.4096>

2 Anders, S. & Huber, W. Differential expression analysis for sequence count data. *Genome Biol* **11**, R106 (2010). <https://doi.org:10.1186/gb-2010-11-10-r106>

3 Wilcoxon, F. Individual comparisons of grouped data by ranking methods. *J Econ Entomol* **39**, 269 (1946). <https://doi.org:10.1093/jee/39.2.269>

4 Dobin, A., Davis, C. A., Schlesinger, F., Drenkow, J., Zaleski, C., Jha, S. *et al.* STAR: ultrafast universal RNA-seq aligner. *Bioinformatics* **29**, 15-21 (2013). <https://doi.org:10.1093/bioinformatics/bts635>

5 Liao, Y., Smyth, G. K. & Shi, W. featureCounts: an efficient general purpose program for assigning sequence reads to genomic features. *Bioinformatics* **30**, 923-930 (2014). <https://doi.org:10.1093/bioinformatics/btt656>

6 Love, M. I., Huber, W. & Anders, S. Moderated estimation of fold change and dispersion for RNA-seq data with DESeq2. *Genome Biol* **15**, 550 (2014). <https://doi.org:10.1186/s13059-014-0550-8>

7 Zhou, Y., Zhou, B., Pache, L., Chang, M., Khodabakhshi, A. H., Tanaseichuk, O. *et al.* Metascape provides a biologist-oriented resource for the analysis of systems-level datasets. *Nat Commun* **10**, 1523 (2019). <https://doi.org:10.1038/s41467-019-09234-6>

8 Hole, P. S., Zabkiewicz, J., Munje, C., Newton, Z., Pearn, L., White, P. *et al.* Overproduction of NOX-derived ROS in AML promotes proliferation and is associated with defective oxidative stress signaling. *Blood* **122**, 3322-3330 (2013). <https://doi.org:10.1182/blood-2013-04-491944>

9 Rapin, N., Bagger, F. O., Jendholm, J., Mora-Jensen, H., Krogh, A., Kohlmann, A. *et al.* Comparing cancer vs normal gene expression profiles identifies new disease entities and common transcriptional programs in AML patients. *Blood* **123**, 894-904 (2014). <https://doi.org:10.1182/blood-2013-02-485771>

10 Svendsen, J. B., Baslund, B., Cramer, E. P., Rapin, N., Borregaard, N. & Cowland, J. B. MicroRNA-941 Expression in Polymorphonuclear Granulocytes Is Not Related to Granulomatosis with Polyangiitis. *PLoS One* **11**, e0164985 (2016). <https://doi.org:10.1371/journal.pone.0164985>

11 Kohlmann, A., Kipps, T. J., Rassenti, L. Z., Downing, J. R., Shurtleff, S. A., Mills, K. I. *et al.* An international standardization programme towards the application of gene expression profiling in routine leukaemia diagnostics: the Microarray Innovations in LEukemia study prephase. *Br J Haematol* **142**, 802-807 (2008). <https://doi.org:10.1111/j.1365-2141.2008.07261.x>

12 Bagger, F. O., Kinalis, S. & Rapin, N. BloodSpot: a database of healthy and malignant haematopoiesis updated with purified and single cell mRNA sequencing profiles. *Nucleic Acids Res* **47**, D881-D885 (2019). <https://doi.org:10.1093/nar/gky1076>
